# Mild concussion impairs extinction of avoidance and alters respective brain circuits in male rats

**DOI:** 10.1016/j.expneurol.2026.115734

**Published:** 2026-03-14

**Authors:** Osmarie Martínez-Guzmán, Mauricio Cáceres-Chacón, Melissa Rivera-López, Gabriela Hernández-Busot, José Forty-Díaz, Héctor G. Haddock-Martínez, Demetrio Sierra-Mercado

**Affiliations:** a Department of Anatomy & Neurobiology, University of Puerto Rico School of Medicine, San Juan 00936-5067, Puerto Rico; b Department of Microbiology & Zoology, University of Puerto Rico School of Medicine, San Juan 00936-5067, Puerto Rico

**Keywords:** Traumatic brain injury, Post-traumatic stress disorder, Gut-brain axis, Gut dysbiosis, Conditioning, Context fear, Rodent

## Abstract

Concussive brain injury is a risk factor for anxiety disorders. Pre-clinical models demonstrate that concussion increases passive fear responses, such as conditioned freezing, yet provide limited insight to active responses like avoidance of perceived threats. This is important because persistent avoidance is characteristic of anxiety disorders. Moreover, brain injury can induce an imbalance of the gut microbiome, which can alter emotions. Adult male rats were trained on a platform-mediated avoidance task where they learned to step onto a platform to avoid a foot shock following a conditioned auditory tone. A sucrose reward was provided via a lever press that is opposite to the platform. Next, closed head injury was delivered to produce a mild concussion. After recovery, separate cohorts of rats were tested to dissociate between changes in avoidance expression and extinction-related processes. Cellular activity was assessed using c-Fos immunohistochemistry in brain regions implicated in avoidance: amygdala, medial prefrontal cortex, insular cortex, ventral striatum, and ventral hippocampus. Fecal pellets were collected to extract genetic material to identify potential changes in populations of bacteria in the gut microbiome. Closed head injury induced persistent avoidance by impairing extinction. Injured rats showed decreased activity in the basomedial amygdala and the CA1 subregion of the ventral hippocampus, increased activity in the rostral insular cortex and ventral striatum, and no change in the medial prefrontal cortex. Closed head injury did not induce changes in gut microbiota. Understanding mechanisms of concussion-induced avoidance is crucial for developing rehabilitation strategies for mental health disorders impacted by brain injury.

## 1. Introduction

Concussive brain injury is a risk factor for fear and trauma-related disorders (Hoge et al., 2014; Hoge et al., 2008; Taylor et al., 2012). Concussion can be produced in rodents via closed head injury. In the closed head brain injury model of concussion, an unhindered downward head acceleration may induce cognitive deficits without gross observable brain damage (Gennarelli et al., 1982; Khuman et al., 2011; Meehan et al., 2012; Witgen et al., 2005; Wu et al., 2022). Closed head injury models can be used to provide insight on how concussion influences fear-related behaviors.

Pre-clinical models have begun to provide a biological link between a single concussion and fear-related behaviors. Several reports have provided conflicting interpretations. Some studies suggest that concussive-injury increases the strength of fear memory (Davies et al., 2016; Meyer et al., 2012; Reger et al., 2012), whereas other studies suggest that concussion decreases fear (Palmer et al., 2016) or has no effect (Cheng et al., 2014; Zhao et al., 2018). A common variable with these studies is that they measured a passive fear response, freezing (Sierra-Mercado et al., 2015b; Sierra-Mercado et al., 2011). Nevertheless, the influence of concussion on active fear responses, specifically avoidance (Aupperle et al., 2012) remains to be elucidated. This is fundamental because persistent avoidance is characteristic of fear and trauma-related disorders (Asmundson et al., 2004; Breslau, 2001).

Avoidance is a form of emotional regulation achieved by evading the source of danger (Mowrer and Lamoreaux, 1946) which can be studied in rodents using the platform-mediated avoidance task (Bravo-Rivera et al., 2014; Diehl et al., 2019). This paradigm consists of a rat stepping onto a platform to avoid a foot shock following an auditory tone that serves as a warning signal. In parallel, food reward is available via a lever that is located opposite to the platform. This creates a conflict where the rat must choose between avoiding the mild foot shock at the expense of food reward. The complexity of the behaviors suggests the involvement of a brain circuit for avoidance (Diehl et al., 2019).

Several brain regions interact to execute different aspects of active avoidance. The amygdala is essential for learning (Lázaro-Muñoz et al., 2010) and expressing avoidance behaviors (Bravo-Rivera et al., 2014; Choi et al., 2010). Furthermore, the ventral striatum is essential for avoidance (Bravo-Rivera et al., 2014; Diehl et al., 2020; Ramirez et al., 2015) and reward seeking (Darvas et al., 2011), whereas the prelimbic medial prefrontal cortex is crucial early in avoidance (Bravo-Rivera et al., 2014; Diehl et al., 2020). Additionally, the insular cortex is implicated in other types of avoidance (Méndez-Ruette et al., 2019; Shin and Liberzon, 2010). Avoidance can be reduced by extinction, whereby presentations of the conditioned stimulus alone (i.e. auditory tone with no shock) reduce time on the platform (Bravo-Rivera et al., 2014). Extinction requires the infralimbic subregion of the prefrontal cortex to inhibit avoidance (Bravo-Rivera et al., 2014; Moscarello and LeDoux, 2013; Rosas-Vidal et al., 2018). Lastly, the ventral hippocampus is involved in the integration of contextual information influencing avoidance behaviors (van der Veldt et al., 2025). Importantly, deficient extinction leads to persistent avoidance (Bravo-Rivera et al., 2015). The influence of concussion on expression and extinction of avoidance is unclear.

Different severities of brain injury can induce dysbiosis of gut microbiota (Celorrio et al., 2021; Ma et al., 2019; Rice et al., 2019; Simon et al., 2020). Dysbiosis of gut microbiota can be harmful for several reasons including the cause of excessive inflammation and disrupted neurotransmitter levels for cognition (Ghaemi and Kheradmand, 2025), which can alter emotional regulation (Needham et al., 2022). This is germane because the gut-microbiota-brain axis influences fear and extinction memories dependent on the amygdala and prefrontal cortex (Chu et al., 2019; Hoban et al., 2018; Hoban et al., 2017; Klarer et al., 2014). In addition, the gut-microbiota-brain axis is necessary for normal function of the striatum (Cox et al., 2024; Han et al., 2018) which is required for avoidance (Han et al., 2018). The influence of concussion on gut microbiota as they relate to avoidance and reward is largely unexplored.

We combined closed head injury as a model of human concussion with platform-mediated avoidance in rats. After recovery from injury, we assessed avoidance in both the presence and absence of the conditioned stimulus. Lastly, we assessed potential changes in gut microbiota. Our results provide evidence that concussion is a risk factor for persistent avoidance.

## 2. Methods

### 2.1. Animals

A total of 46 male Sprague-Dawley rats (Envigo+, Indiana, USA) weighing 300–350 g were housed in individual cages on a 12-h light/12-h dark cycle. Individual housing ensures that each rat can consume its allotted food and prevents stressors related to competition with other rats for the rationed food (Bravo-Rivera et al., 2021; Quirk et al., 2000). All experiments were run between 9 AM and 2 PM. A random number generator was used to assign rats to subgroups that would be either sham-control or closed head injury. In accord with ethical guidelines, all procedures were approved by the Institutional Animal Care and Use Committee (Protocol A120117), which is accredited by the Association for Assessment and Accreditation of Laboratory Animal Care International (AAALAC). Thus, the environmental parameters of luminance, ambient noise, air moisture, and temperature are strictly regulated ensuring premium animal care and welfare at all times. Experiments were performed by investigators blinded to the groups. We performed a power analysis for sample size estimates for groups using the formula: n = (z^2^ × σ^2^)/d^2^, with each of the variables defined as: z = the level of confidence desired in standard deviations, σ = the estimate of the population standard deviation, and d = the acceptable width of the confidence interval. For measurements of platform-mediated avoidance, we use a 0.05 confidence level, and required a confidence interval of 10% of the maximal threat response value. The maximal standard deviation for these data is approximately 6 s. Considering this, preliminary sample size estimates were determined by calculating z = 1.96 (corresponding to confidence of 0.05); σ = 6 s; d = (30*0.10) = 3. Sample size estimates were optimized based on fundamental work of platform-mediated avoidance (Bravo-Rivera et al., 2014), such that each group had 12 animals, which is consistent with most studies that evaluate threat responses elicited by tone-shock associations (Carneiro et al., 2018).

### 2.2. Bar-press training

**Fig. 1.**
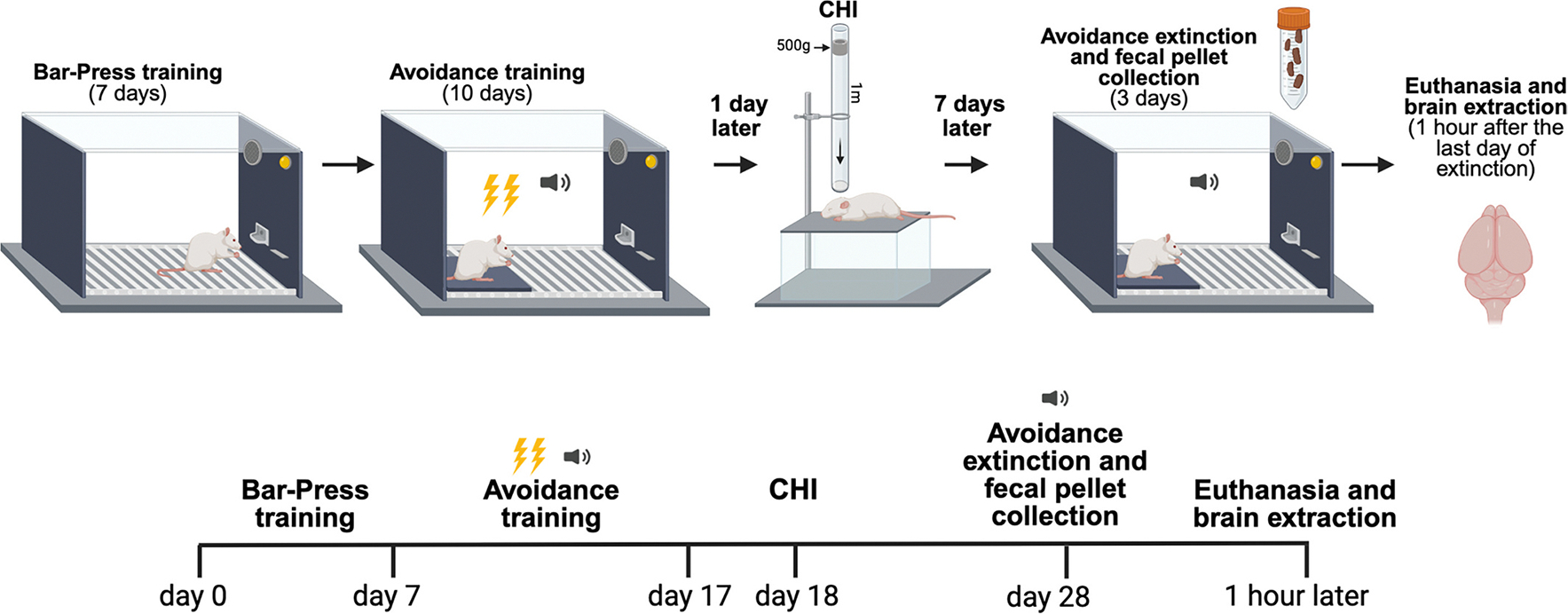
Experimental timeline. Rats underwent bar-press training to establish operant responding for sucrose reward. Rats were then trained on platform-mediated avoidance, during which a tone (conditioned stimulus, CS) predicted a footshock (unconditioned stimulus, US). In parallel, an acrylic platform was positioned opposite from the bar press, permitting avoidance of the foot shock. Stepping on the platform comes at a cost, since the rat cannot press a lever for reward. Next, after avoidance training, anesthetized animals received a closed head injury (CHI) via weight drop. After recovery, animals were returned to the same chamber, during which the tone was presented without the shock for either avoidance test (Sham: *n* = 11; CHI: *n* = 11) or extinction (Sham: *n* = 12; CHI: *n* = 12). Lastly, animals were euthanized and brains were extracted for further analysis.

Prior to avoidance conditioning, rats were food-restricted to 18 g per day of standard rat chow (Fig. 1). Food restriction occurred as part of the training for rats to learn to press a bar for sucrose pellets (Sierra-Mercado et al., 2006). Bar-press training took place on a variable interval schedule of reinforcement (VI-30) by which the animal receives one pellet approximately every 30 s regardless of the number of presses. Rats were trained until they performed at 10 presses/min. Food access was returned to *ad libitum*. Food restriction was temporary and lasted for approximately 7 days. We highlight that the short-term food restriction during bar-press training was mild and insufficient to induce biological benefits related to calorie restriction (Ingram and de Cabo, 2017). Accordingly, potential concerns related to calorie restriction affecting behavior, being neuroprotective around brain injury, or influencing biological measures were assuaged (Zhang et al., 2021).

### 2.3. Platform-mediated active avoidance

Avoidance training occurred in the same chamber in which rats learned to bar-press for sucrose pellets, as described previously (Bravo-Rivera et al., 2014). Briefly, rats were placed in the operant chamber that was inside a sound-attenuating box (Med-Associates). The chamber contained a square, acrylic platform 14.50 cm × 14.50 cm positioned opposite from the bar-press. Rats were delivered 10 conditioning trials pairing the tone (4 kHz, 75 dB, 30 s, 3 min intertrial interval) with a mild, scrambled foot shock (0.4 mA, 2 s) that co-terminates with the tone per day, for 10 days. Stepping on the platform comes at a cost, since the rat cannot press a lever for reward.

### 2.4. Closed head injury

**Fig. 2.**
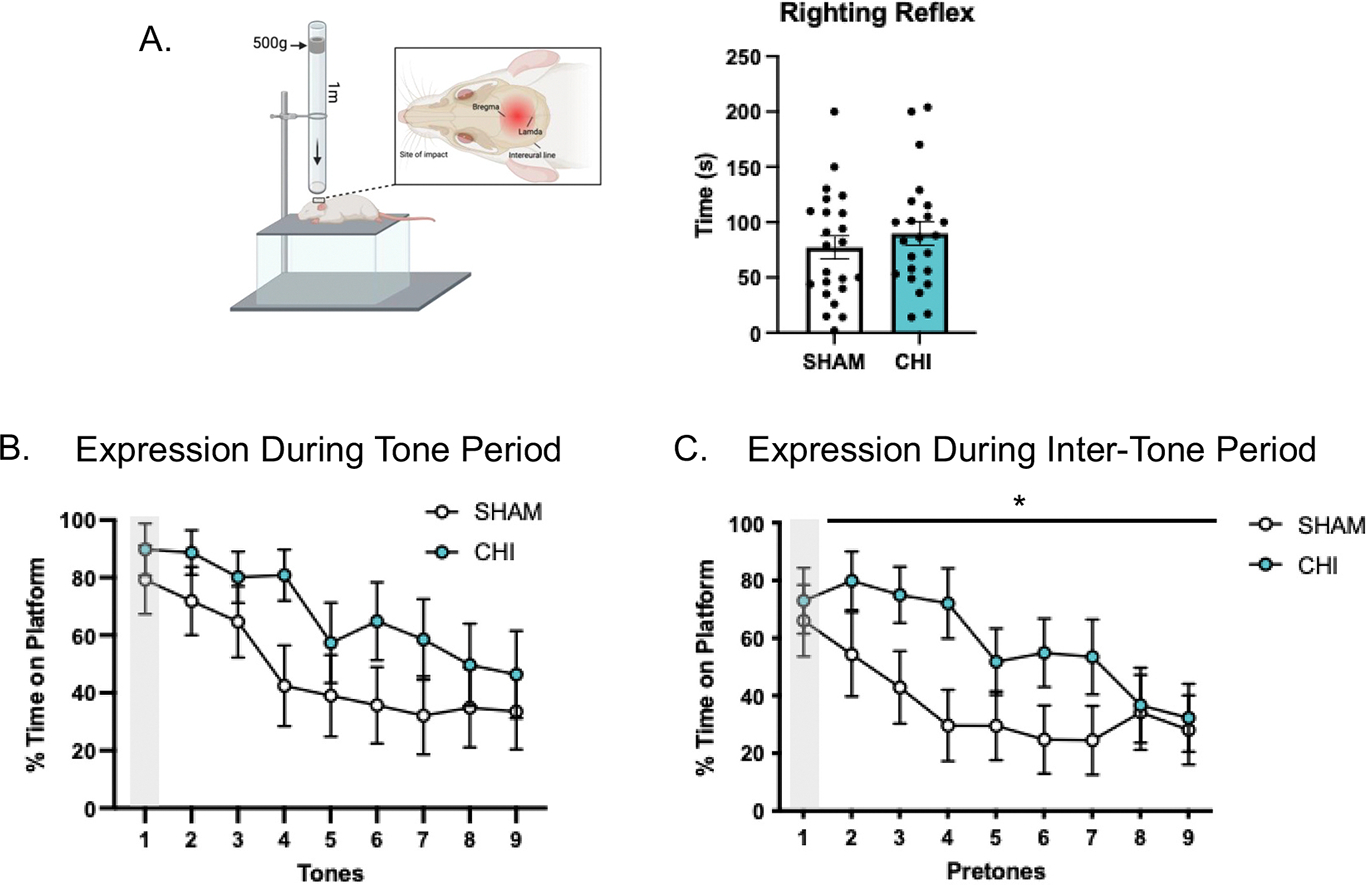
Effects of Closed Head Injury (CHI) on expression of avoidance. A) CHI impact is administered between the lambda and bregma points on the skull. There was no difference in time to display righting reflex following CHI (SHAM: *n* = 23; CHI: *n* = 23; t_44_: 0.8613; *p* = 0.3937). Thus, injury level was consistent with mild concussive brain injury. B) CHI does not influence the percent time spent on the platform during the tone period (ANOVA, *p* = 0.624). C) CHI induces excess avoidance during the intertone (i.e. safe) period (ANOVA, *p = 0.0467). Shaded regions indicates comparable levels of avoidance, indicating that CHI does not alter the strength of avoidance.

Closed head injury was achieved using a weight drop apparatus (Fig. 2A) that was built in the laboratory ensuring that weight-drop did not cause fractures, lesions, or hemorrhage (Henninger et al., 2005; Meyer et al., 2012; Mychasiuk et al., 2014; Weber, 2007). The technique involves a PVC guide tube with holes drilled to minimize friction and air resistance to the weight (Davies et al., 2016; Henninger et al., 2005; Paxinos and Charles Watson, 2007). A cylindrical weight (500 g; 9-cm diameter; densely packed, powdered lead, GolfWorks, Newark, OH) was placed in the PVC guide tube. The weight was tied to a cable to ensure that there was no rebound of the weight upon impact. Rats were anesthetized using 4% isoflurane and oxygen for two minutes. The animal’s head was shaved, and the interaural line was marked with a non-toxic pen. The animal was removed from the isoflurane and immediately placed perpendicular under the tube in the prone position. The posterior portion of the PVC guide tube was placed on the interaural line to ensure consistent weight drops and replicable injury between rats. Placement of the tube on the interaural line indicates that the weight targeted between lambda and bregma, which are separated by 9 mm (Paxinos and Charles Watson, 2007). The weight was dropped from a height of 1 m by pulling a stop bolt from the tube that was holding the weight. At impact, there was unhindered, downward head acceleration (Witgen et al., 2005). The animal landed on cushions located below to prevent bodily injury. The rat was given an analgesic within a few seconds before weight drop (i.e., meloxicam 5 mg/kg i.m.). Sham injury involved each handling step and isoflurane exposure, except for the weight drop. Time to display righting reflex from the back to limbs was recorded and served as an index of loss of consciousness (Berman et al., 2023) (Fig. 2B).

### 2.5. Open field test

Rats were placed in the center of an open field (100 × 100 cm) for 5 min to measure locomotion and exploratory behavior. The open field is divided into peripheral and central regions of equal area (Mueller et al., 2010). Distance traveled was measured to determine locomotion, and the time spent in the center is used to determine anxiety-like behavior. Time in the center and distance traveled were recorded and analyzed. We assessed the influence of closed head injury on behaviors in the open field at two time points in separate cohorts of animals: 1) 1 h after injury, and 2) 6 days after injury.

### 2.6. Elevated plus maze

Rats were placed in an elevated plus maze for 5 min to measure risk assessing behaviors. The maze was raised 79 cm from the floor and consisted of a “plus-sign shaped” acrylic platform. Two of the arms were 50 × 10 × 48 cm and were closed with opaque walls, whereas the other two arms were 50 × 10 cm, and were open with short, transparent walls of 3.8 cm that permit vision and proprioception to trigger aversion to the open spaces (Martínez et al., 2002). The arms were arranged perpendicular to each other, with a central neutral area of 10 × 10 cm connecting them. Rats were placed in the center of the maze facing one of the open arms. The time spent in the arms was recorded and analyzed. We assessed the influence of closed head injury on behaviors in the elevated plus maze 1 week after injury.

### 2.7. Expression and extinction of platform-mediated active avoidance

**Fig. 3.**
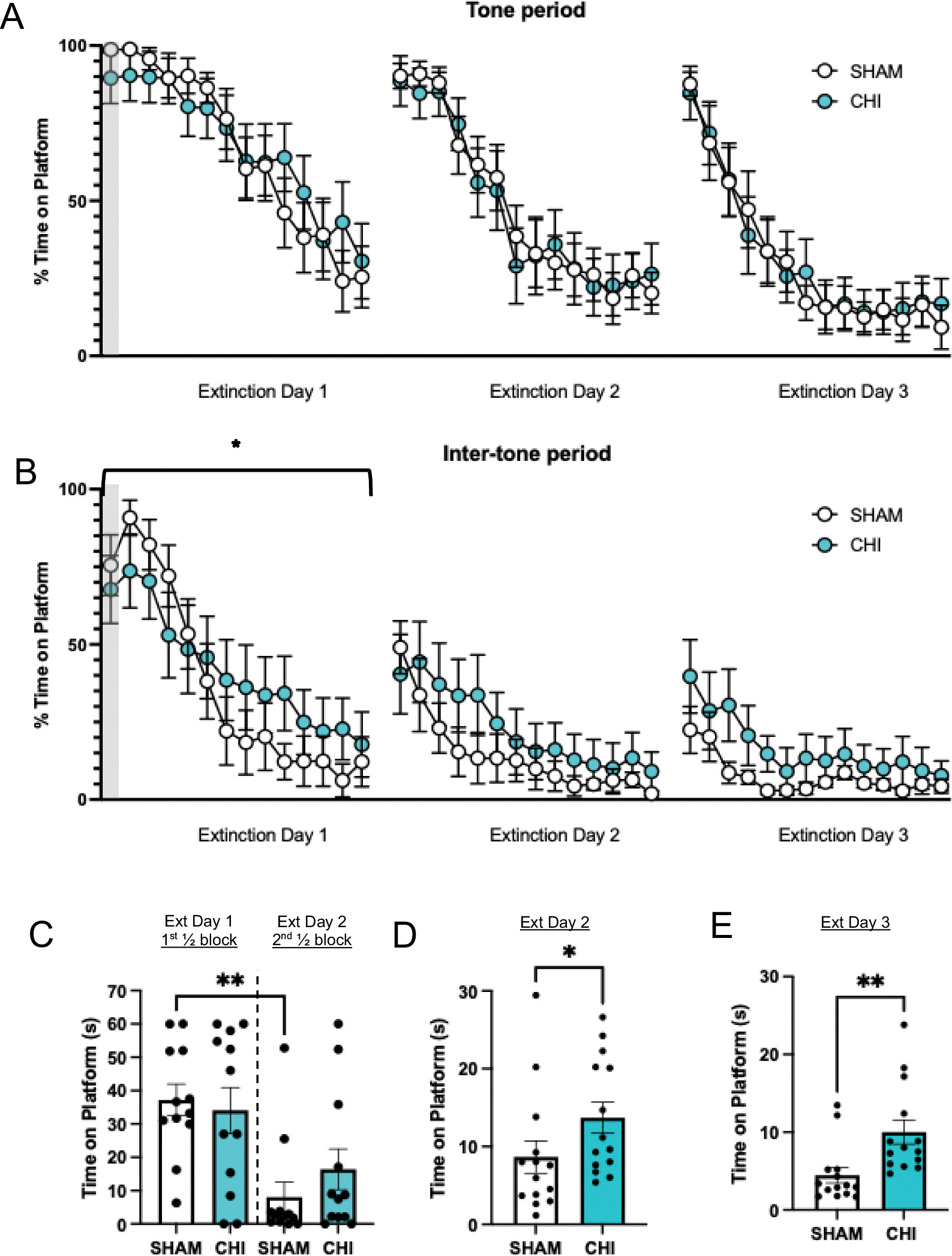
Effects of Closed Head Injury (CHI) on extinction of avoidance. A) CHI does not affect time spent on the platform during the tone (immediate threat) across extinction. B) However, during the inter-tone period (no threat), CHI rats spent more time on the platform on Extinction Day 1 (ANOVA, *p < 0.0127). C) Data from Fig. 3B were collapsed for Extinction Day 1 for the inter-tone period. For within-session extinction, SHAM controls displayed reduced avoidance during the late phase (second ½ block of trials) of extinction as compared to the early phase (first ½ block of trials) of extinction (one-way ANOVA, *p* = 0.0038). On the contrary, CHI rats did not display reduced avoidance during the late phase of extinction (one-way ANOVA, *p* = 0.137). D) Data from Fig. 3B were collapsed for Ext Day 2, and E) Ext Day 3. On either day, time on platform was higher in CHI animals compared to SHAM (Student’s *t*-test, one-tailed, *p* < 0.05). Data are presented as mean ± SEM. SHAM = sham-injured animals; CHI = closed head injury. (Sham: *n* = 12; CHI: *n* = 12.). Shaded regions indicate comparable levels of avoidance, highlighting that CHI does not alter the strength of avoidance.

**Fig. 4.**
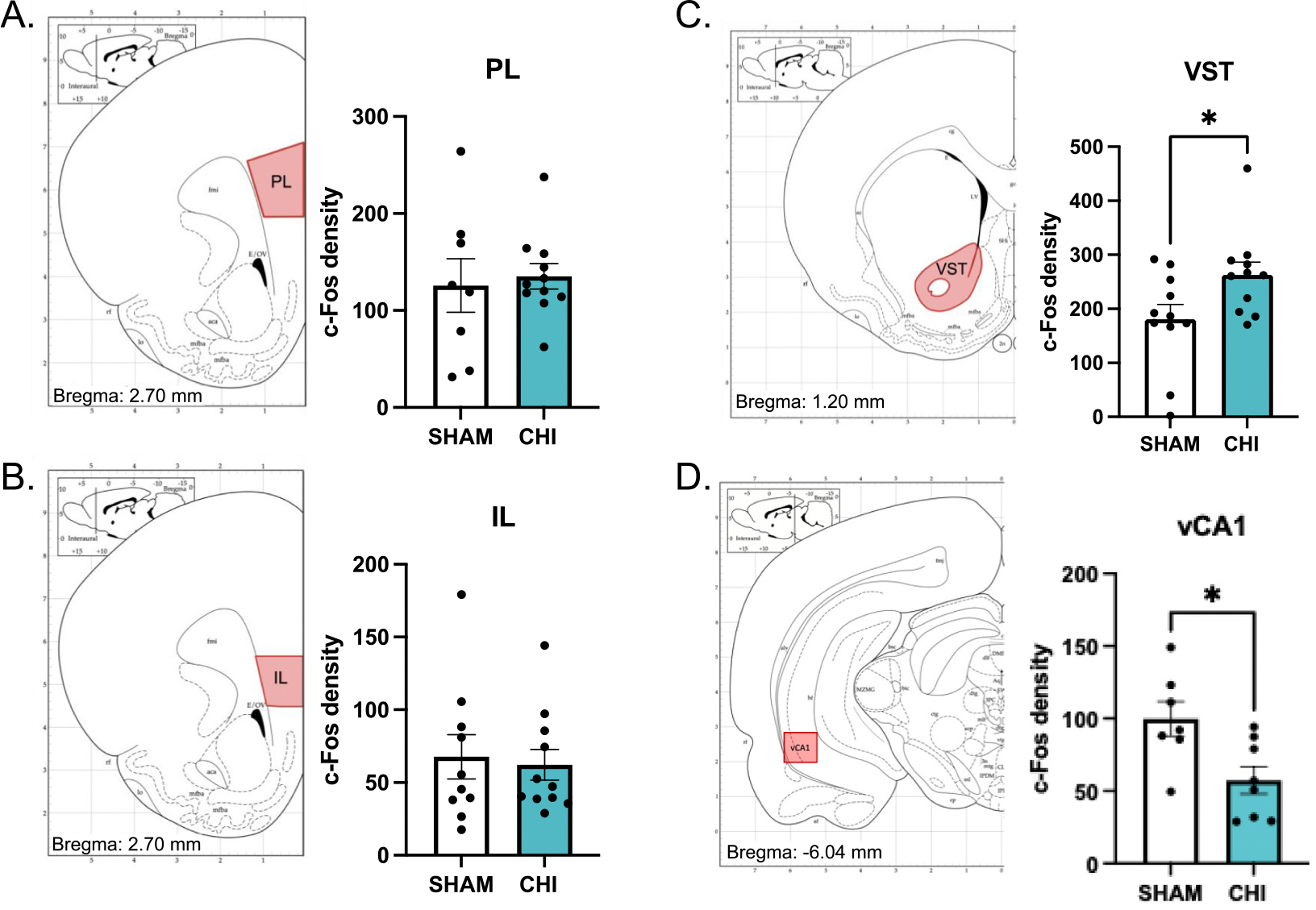
Neural correlates of brain regions implicated in avoidance for rats that were extinguished on avoidance shown on Fig. 3. We did not observe differences in c-Fos labeling in either A) Prelimbic (PL) cortex, or B) Infralimbic (IL) cortex. We observed increases in c-Fos labeling in C) the Ventral Striatum (VST; *p = 0.0346) and decreased labeling in D) the CA1 region of the ventral hippocampus (vCA1; *p = 0.0147). The increased c-Fos labeling in VST and decreased labeling in vCA1 correspond to the increased avoidance observed in the absence of the threatful stimulus (i.e. inter-tone period).

The day following testing in the elevated plus maze (i.e. 1 week following closed head injury (CHI)), rats were placed back in the conditioning chamber where they were trained on avoidance. We performed two sets of experiments to compare expression of avoidance and extinction behaviors. For tests of avoidance expression, rats (CHI: *n* = 11; Sham: *n* = 11; Fig. 3) were delivered 9 trials of tone alone (no shock). After presentation of the last tone, brain extraction was performed as described below. To assess for extinction of avoidance, a separate cohort of animals (CHI: *n* = 12; Sham: *n* = 12, Fig. 4) was delivered 14 trials of tone alone (no shock) per day, for three days, followed by brain extraction. Overall, this approach allowed us to dissociate between expression and extinction related processes in analyses of brain tissue.

We assessed avoidance behavior in two conditions: 1) during the tone period when the conditioned stimulus is present (Figs. 2B & 3A), representing threat, and 2) after offset of the tone during the inter-tone period (Figs. 2C & 3B). Conceivably, the inter-tone period is when there is absence of the threat, making this the safest period of the session. This allowed us to compare influences of fear (i.e. presence of threat) and anxiety (absence of threat) on avoidance.

### 2.8. Immunohistochemistry and c-Fos quantification

Only brain sections with regions of interest that were clearly identifiable were included in the analyses. To ensure consistency across animals, regions of interest were identified by matching brain sections according to landmarks and cytoarchitecture in the stereotaxic atlas (Paxinos and Charles Watson, 2007). Counting for c-Fos positive cells was performed from images taken from both hemispheres for at least two brain slices. Counts were made by an experimenter blinded to the conditions and verified by another blinded experimenter. The average counts between the two experimenters was included. Lastly, the brains were decoded with respect to their experimental conditions.

Immunohistochemistry was used to label c-Fos. One-hour after the last auditory tone in extinction of avoidance, rats were deeply anesthetized with an intraperitoneal injection of pentobarbital sodium (450 mg/kg) and 0.1 ml of heparin (400UI/Kg; Sigma Aldrich H3393–100KU) and euthanized according to the American Veterinary Medical Association (AVMA) guidelines (Leary et al., 2020). Afterwards, rats were transcardially perfused with 250 ml of 0.9% saline solution followed by 500 ml of 4% paraformaldehyde and 0.1 M sodium phosphate buffer at a pH of 7.4. Brains were removed and stored in a 20% sucrose and potassium phosphate buffer (KPBS) for 24 h and placed in a 30% sucrose and KPBS solution for cryoprotection.

Brains were coronally sectioned in slices of 40 μm at the level of the medial prefrontal cortex [mPFC, (AP: 2.76 mm to 3.24 mm)], basomedial and basolateral amygdala [BMA and BLA; AP: −1.72 mm to −2.40 mm)], rostral insular cortex [ICr; AP: 0.24 mm to 0.00 mm], caudal insular cortex [ICc; AP: 1.80 mm to 2.04 mm], and ventral hippocampus [vHPC; AP: −5.60 mm to −6.04 mm] using a cryostat (CM 1850; Leica Biosystems Inc., Buffalo Grove, IL). Brain slices were placed in wells of anti-freeze solution (sucrose, NaPBS, and ethylene glycol) to be used for immunohistochemistry.

To assess c-Fos immunostaining, brain slices were blocked first in a 0.01% triton (Triton X-100, Sigma-Aldrich^®^, USA), 10% normal goat serum (NGS, Vector Laboratories^®^, USA) and 0.12 M potassium buffer saline solution for an hour. Thereafter, brain slices were incubated overnight in rabbit anti-phospho-c-fos (Cell signaling; #5348) at room temperature at a dilution of 1:1000. This was followed by an hour of incubation at room temperature in a solution of biotinylated goat anti-rabbit immunoglobulin G (Vector Laboratories) and placed in a mixed avid-biotin horseradish peroxidase complex solution (ABC Elite Kit; Vector Laboratories) for 30 min. Black immunoreactive nuclei labeled for c-Fos were visualized after 2–3 min of exposure to DAB/peroxidase substrate kit (Vector Laboratories). Sections were mounted on gelatin-coated slides, allowed to dry, covered with a drop of anti-fade mounting media (Vectashield Vector Laboratories^®^, USA) and cover slipped.

### 2.9. Immunohistochemistry analysis

c-Fos labeled cells were counted at 10× magnification using a Nikon microscope (Model Eclipse Ni-E). Micrographs were generated for each region of interest. c-Fos labeled cell counts were averaged for each hemisphere in one section for each brain region using NIS-Elements BR 5.30.05 software. Density was calculated by dividing the number of c-Fos positive cells by the total area of each region as described previously (Cáceres-Chacón et al., 2025).

### 2.10. DNA Extraction from fecal boli and 16S sequence analysis

In the cohort of animals that underwent extinction for three days, fecal pellets were collected and 200 mg were used for gDNA extraction by closely adhering to the instructions provided in the commercially available kit (DNeasy PowerSoil Pro Kit, QIAGEN, Germantown, MD, USA). Raw samples were processed by an external laboratory where the 16S rRNA gene was amplified with universal 16S V4 as per the earth microbiome protocol and sequenced using Illumina MiSeq (Louisiana State Health Sciences Center). Raw fastq files were uploaded as study ID 14456 to Qiita (Gonzalez et al., 2018), and the 16S rRNA sequencing data can be accessed from the European Nucleotide Archive under the study accession number PRJEB108858 (ERP189693). Sequences of the 16S gene V4 region were analyzed in QIIME and filtered for quality and chimeras (using Usearch61). MicrobiomeAnalyst was used to compute the alpha diversity measurement (Chao1 index) and beta diversity on a rarefied operational taxonomic unit (Kim et al., 2017; Knight et al., 2018; Shannon, 1948; Su, 2021).

### 2.11. Data analyses

Time on platform was quantified as a percent during either the 30 s tone presentation or 60 s period after the tone. Commercially available software was used to record and analyze videos (ANY-maze, Stoelting, Wood Dale, IL). The D’Agostino & Pearson Test was used to verify the normal distributions of behavioral data. Platform data were analyzed using repeated-measures analyses of variance (ANOVA), with trial and group as dependent variables (Prism, La Jolla, CA). We assessed for extinction in two stages: within-session extinction and between-session extinction (Plendl and Wotjak, 2010). For within-session extinction, we also compared the early phase of extinction (first ½ block of trials) of the session with the late phase of extinction (second ½ block of trials) (Jiang and Greening, 2021; Stout et al., 2018; Watanabe et al., 2021). For between session extinction across days, data were collapsed and analyzed with a Student’s *t*-test. Distance traveled in the open field and time spent in the open arms of the elevated plus maze were recorded and analyzed with Student’s t-test. Number of c-Fos labeled cells and alpha diversity of gut microbiome data were analyzed with Student’s t-test, whereas beta diversity of gut microbiome data were analyzed with permutational analysis of variance (PERMANOVA). Experimenters performing analyses of all behavioral and histological data were blinded to the condition of the treatment group to minimize bias. Data are presented as the mean ± standard error of the mean (S.E.M.). Differences were considered significant when corresponding *p* values were ≤0.05.

## 3. Results

### 3.1. Closed head injury mimics mild concussion

Single hit, closed head injury (CHI; Fig. 2A) did not influence the time in righting reflex (SHAM: 77.13 s, CHI: 89.91 s; t_44_ = 0.86, *p* = 0.39, Fig. 2A), consistent with a mild level of concussive injury (Berman et al., 2023).

### 3.2. Closed head injury has no effect on locomotion or anxiety-like behaviors

We assessed the influence of closed head injury on locomotion using the open field test at two time points in separate cohorts of animals. Distance traveled at one hour after injury was indistinguishable between groups (SHAM: 33.77 m, CHI: 33.52 m, t_22_ = 0.117, *p* = 0.908), as well as at 24 h (SHAM: 20.20 m, CHI: 23.96 m; t_20_ = 0.892, *p* = 0.388). A lack of difference in locomotor behavior supports a mild level of concussion. Subsequently, after 1 week of recovery, we assessed the effect of closed head injury on anxiety-like behaviors in the elevated plus maze. Closed head injury did not affect anxiety-like behaviors, as indicated by time spent in the open arms of the elevated plus maze (SHAM: 37.62 s, CHI: 43.09 s; t_44_ = 0.639, *p* = 0.526; Supplementary Fig. 2).

### 3.3. Acquisition of platform-mediated avoidance

All rats acquired the ability to express platform-mediated avoidance by stepping onto the platform at the start of the tone (Supplementary Fig. 1) and returning to the bar-press at the end of the tone (Bravo-Rivera et al., 2014). One week after avoidance training, rats were delivered closed head injury.

### 3.4. Closed head injury does not influence the strength of avoidance memory, but impairs extinction

A week following closed head injury, rats were tested for the strength of avoidance memory and extinction. First, to assess the strength of avoidance memory, percent time on the platform during the first tone was similar between sham controls and closed head injury (SHAM: 79.27%; CHI: 89.80%, t_20_ = 0.704, *p* = 0.490, Fig. 2B, shaded region) as well as the first pre-tone period (SHAM: 66.05%; CHI: 72.94%, t_20_ = 0.409 *p* = 0.687, Fig. 2C, shaded region). This observation was replicated in the other cohort of animals as the percent time on the platform during the first tone was identical between sham controls and closed head injury (SHAM: 98.67%; CHI: 89.5%, t_22_ = 1.13, *p* = 0.278, Fig. 3A, shaded region) and first pre-tone period (SHAM: 75.48%; CHI: 67.65%, t_22_ = 0.533, *p* = 0.599, Fig. 3B, shaded region). Together, these data suggest that closed head injury does not influence the strength of avoidance memory.

We examined the effects of closed head injury on within-session extinction. Here, we observed that closed head injury animals spent similar amounts of time on the platform as SHAM-controls during the tone period (F_8, 160_ = 0.776; *p* = 0.624, Fig. 2B). Remarkably, as we assessed for within-session extinction during the inter-tone period, closed head injury animals spent more time on the platform compared to SHAM controls (F_8, 160_ = 2.024; *p* = 0.0467, Fig. 2C). Notably, we observed the same pattern of effects in the other cohort of animals, where closed head injury animals spent more time on the platform during the inter-tone period compared to SHAM controls (F_13, 286_ = 2.130; *p* = 0.0127, Fig. 3B), but not the tone period (F_13, 286_ = 1.179; *p* = 0.294, Fig. 3A), on Extinction Day 1. Likewise, sham controls displayed less time on the platform during the inter-tone period of the late phase (second ½ block of trials) of the extinction session as compared to the early phase (first ½ block of trials) of the session (SHAM early phase: 37.20 s; SHAM late phase: 8.06 s, F_3,44_ = 6.129, *p* = 0.0038, Tukey’s post hoc, Fig. 3C, ANOVA), supporting extinction in sham controls. Contrary to this, animals that received closed head injury did not display a reduction in time on the platform during the late phase of the session compared to the early phase (CHI early phase: 34.07 s; CHI late phase: 16.41 s, F_3,44_ = 6.129, *p* = 0.137, Fig. 3C), indicating deficient extinction. Next, for between session extinction which we assessed in the separate cohort of animals that was given additional days of extinction, time on platform was the same in both sham and injury groups during the tone for Extinction Day 2 (F_13, 286_ = 0.348; *p* = 0.984) and Day 3 (F_13, 286_ = 0.278; *p* = 0.994, Fig. 3A). Of note, as predicted based on the observation with the other cohort of animals, further analyses revealed that closed head injury rats spent more time on the platform during the inter-tone period compared to SHAM controls (SHAM: 8.651 s, CHI: 13.72 s; t_26_ = 1.756, *p* = 0.0454; Student’s *t*-test, one-tailed, Fig. 3D), which persisted on extinction day 3 (SHAM: 4.468 s, CHI: 10.01 s; t_26_ = 3.014, *p* = 0.0028; Student’s t-test, one-tailed, Fig. 3E).

### 3.5. Closed-head injury alters neural activation within the threat-avoidance circuit in rats that underwent extinction

To examine whether closed head injury alters neuronal activation in regions involved in avoidance behavior and threat interpretation, we quantified c-Fos expression (Supplementary Fig. 4) in the prelimbic cortex (PL), infralimbic cortex (IL), ventral striatum (VST), basomedial amygdala (BMA), basolateral amygdala (BLA), and the insular cortex (IC) following the platform-mediated avoidance task.

We quantified c-Fos expression in brain regions implicated in the expression of platform-mediated avoidance (Bravo-Rivera et al., 2015; Bravo-Rivera et al., 2014). To achieve this, we focused on prefrontal and striatal regions in the cohort of animals that underwent test for avoidance expression, but not extinction over days. c-Fos density analysis revealed no group differences in the prelimbic (SHAM: 289.5 counts/cm^2^, CHI: 199.3 counts/cm^2^; t_15_ = 1.290, *p* = 0.217; Supplementary Fig. 3A) and infralimbic cortices (SHAM: 218.3 counts/cm^2^, CHI: 159.9 counts/cm^2^; t_14_ = 1.036, *p* = 0.318, Supplementary Fig. 3B), or the ventral striatum (SHAM: 124.0 counts/cm^2^, CHI: 146.5 counts/cm^2^; t_16_ = 0.585, *p* = 0.567, Supplementary Fig. 3C). Then, we assessed the hippocampus given its implication in anxiety and avoidance. c-Fos labeling in the CA1 region of the ventral hippocampus (vCA1) was decreased in closed head injury animals (SHAM: 87.99 counts/cm^2^, CHI: 32.4 counts/cm^2^; t_15_ = 2.459, *p* = 0.0266, Supplementary Fig. 3D), but not in vCA2 (SHAM: 75.1 counts/cm^2^, CHI: 62.39 counts/cm^2^, *p* = 0.673) or vCA3 (SHAM: 40.8 counts/cm^2^, CHI: 22.14 counts/cm^2^, *p* = 0.380) subregions.

**Fig. 5.**
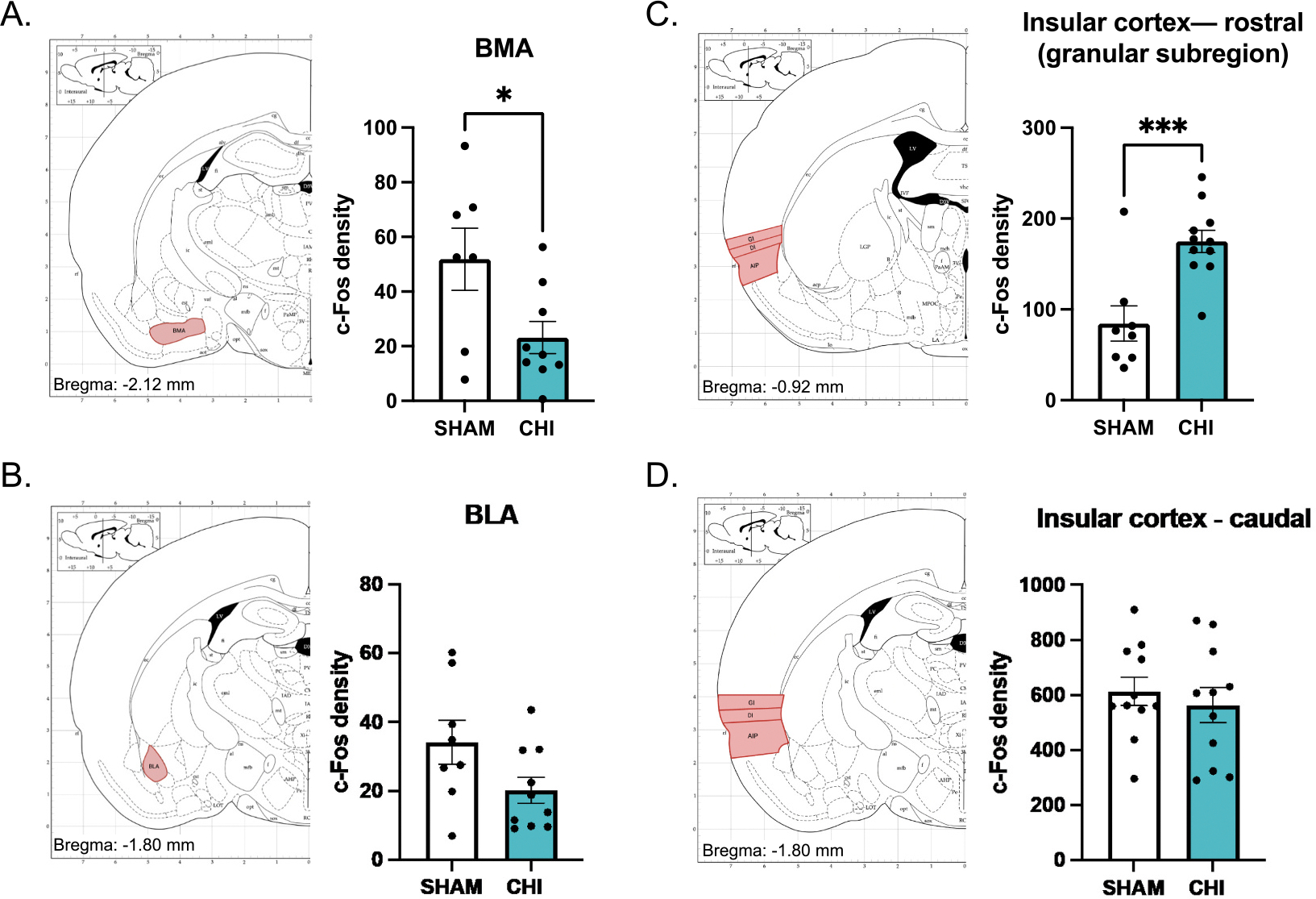
Neural correlates of brain regions implicated in anxiety. A) Increased in c-Fos labeling in the basal medial amygdala (BMA; *p = 0.0312), but not B) the basolateral amygdala (BLA). C) Increased c-Fos labeling was observed in the rostral portion of the insular cortex (*p = 0.0007), but not the D) caudal portion of the insular cortex.

In the separate cohort of animals that underwent extinction over days, there were no differences in c-Fos density between closed head injury and Sham animals in the PL (SHAM: 127.5 counts/cm^2^, CHI: 135.2 counts/cm^2^; t_17_ = 0.342, *p* = 0.737, Fig. 4A) or IL (SHAM: 67.69 counts/cm^2^, CHI: 62.19 counts/cm^2^; t_19_ = 0.300, *p* = 0.767, Fig. 4B). On the other hand, c-Fos density in the VST was increased in closed head injury animals compared to SHAM controls (SHAM:180.7 counts/cm^2^, CHI: 262.8 counts/cm^2^; t_20_ = 2.268, *p* = 0.0346, Fig. 4C). Moreover, within the amygdala, we observed a reduction in c-Fos density in the BMA of closed head injury animals compared to sham (SHAM: 51.83 counts/cm^2^, CHI: 23.14 counts/cm^2^; t_14_ = 2.395, *p* = 0.0312), Fig. 5A, whereas BLA c-Fos density was not reduced (SHAM: 34.05 counts/cm^2^, CHI: 20.21 counts/cm^2^; t_16_ = 1.95, *p* = 0.0685, Fig. 5B). We separated the analyses of the insular cortex (IC) into the rostral and caudal subdivisions. In the rostral granular subregion of the IC, closed head injury animals exhibited a marked increase in c-Fos density compared to sham controls (SHAM: 84.45 counts/cm^2^, CHI: 174.9 counts/cm^2^; t_17_ = 4.14, *p* = 0.0007, Fig. 5C). The caudal insula showed no group differences (SHAM: 612.9 counts/cm^2^, CHI: 563.3 counts/cm^2^; t_20_ = 0.604, *p* = 0.553, Fig. 5D). Lastly, c-Fos labeling was decreased in the CA1 region of the ventral hippocampus (vCA1) in closed head injury animals (SHAM: 99.8 counts/cm^2^, CHI: 57.3 counts/cm^2^; t_13_ = 2.812, *p* = 0.0147, Fig. 4D), but not in vCA2 (SHAM: 48.70 counts/cm^2^, CHI: 63.42 counts/cm^2^, *p* = 0.528) or vCA3 (SHAM: 63.45 counts/cm^2^, CHI: 27.81 counts/cm^2^, *p* = 0.323) subregions.

### 3.6. Closed head injury does not influence gut microbiota diversity and composition

**Fig. 6.**
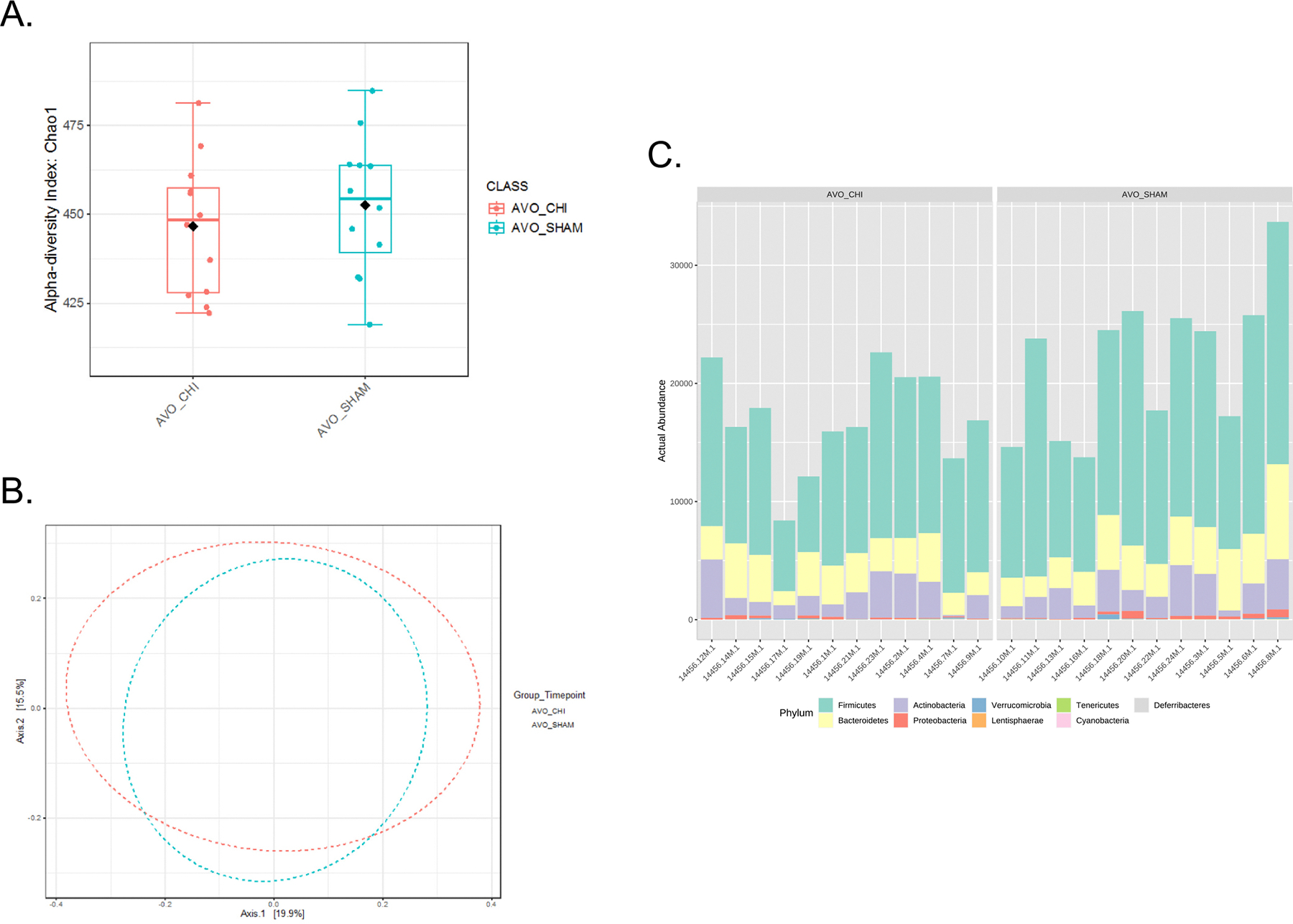
Effects of Closed Head Injury (CHI) on gut microbiota diversity and relative abundance from animals delivered extinction. CHI does not affect A) alpha diversity (*p* = 0.44216, t-test) or B) beta diversity (*p* = 0.758, F-value: 0.76694; PERMANOVA). C) CHI does not affect the phylogenetic classification of fecal bacteria communities at the phylum level. (SHAM: *n* = 12; CHI: *n* = 12).

We evaluated the effects of closed head injury on gut microbiota composition. To achieve this, we assessed alpha diversity to gain insight into the number of different species and their relative abundance, and beta diversity to understand the comparison between populations of bacterial taxa at the phylum level. Analysis of alpha diversity using the Chao1 index revealed no differences between closed head injury and Sham animals, suggesting comparable species richness across groups (Fig. 6A, *p* = 0.442). Beta diversity analysis via Principal Coordinates Analysis (PCoA) did not demonstrate distinct clustering between groups, indicating that the overall microbial community structure remained largely unaffected by closed head injury (Fig. 6B, *p* = 0.758). Similarly, the relative abundance of dominant bacterial phyla, including Firmicutes, Bacteroidetes, Actinobacteria, and Proteobacteria, was consistent between closed head injury and SHAM groups (Fig. 6C).

## 4. Discussion

We employed a single-hit, closed head injury to mimic mild concussion and assessed platform-mediated avoidance. We observed persistent avoidance in the absence, but not in the presence, of the conditioned stimulus threat. Persistent avoidance was due to impaired extinction. The results have implications for our understanding of how concussion induces persistent avoidance contributing to mental health disorders.

Our closed-head injury model is analogous with mild concussion. In support of this idea, closed head injury had no effect on the time to display the righting reflex, or distance traveled in the open field. Thus, our closed head injury mimicked mild traumatic brain injury (Berman et al., 2023; Henninger et al., 2005). Interestingly, we did not observe changes in anxiety-like behavior in the elevated plus maze. This was in contrast to what was observed by the pioneering work of Forster and colleagues (Davies et al., 2016; Meyer et al., 2012) who reported increased anxiety in the elevated plus maze one week after recovery from closed head injury (Davies et al., 2016; Meyer et al., 2012). A parsimonious explanation is that their work used cohorts of naïve animals in their assessment of the elevated plus maze. Contrary to this, the animals in our study received mild foot shocks during avoidance training that could have altered subsequent behavioral outputs related to anxiety in the elevated plus maze (Grahn et al., 1995; Korte and De Boer, 2003). Notably, the subtle differences in the apparatuses used in their studies and ours (i.e. no walls versus short, transparent walls on the open arms, and slightly different height of the platforms) are not likely the reason for the differences between the studies (Martínez et al., 2002). All things considered, potential group differences in the elevated plus maze may have been masked due to the previous experience of the avoidance training.

The persistent avoidance we observed is likely due to impaired extinction and not a stronger memory of avoidance. We were able to distinguish between these two possibilities. First, we observed that avoidance levels in the initial trials were identical to sham controls, suggesting that closed head injury does not influence the strength of the avoidance memory. In fact, preclinical work in rodents involving Pavlovian fear conditioning measuring freezing suggested that brain injury increases the strength of fear memory. For example, Reger et al. (2012) report that concussive injury enhanced fear expression. However, it should be noted that their freezing data obtained over multiple test trials were collapsed, making it difficult to dissociate between enhanced fear expression and impaired extinction. Hence, increased freezing in their report may be due, at least in part, to impaired extinction.

Persistent avoidance in the absence of the conditioned stimulus after closed head injury points us to the complex neural circuitry underlying behavioral changes. We did not observe changes in activity in either the prelimbic or infralimbic cortices as measured by c-Fos labeling. This was somewhat expected since there was no effect on avoidance during presentation of the threatful auditory stimulus. Next, we assessed brain regions implicated in anxiety, since persistent avoidance occurred in the absence of the auditory stimulus. We focused on the basomedial and basolateral subregions of the amygdala, since both subregions of the amygdala are implicated in different aspects of emotional regulation related to anxiety and fear expression (McDonald, 2020). Briefly, for anatomical perspective, the basomedial amygdala is found in the ventral portion of the amygdala neighboring the basolateral nucleus. In support of this distinction, we observed decreased activity in the basomedial amygdala which is implicated in anxiety (Adhikari et al., 2015), but not the basolateral amygdala which is implicated in conditioned fear responses like freezing (LeDoux and Pine, 2016). Another brain region, the rostral insular cortex, is a driver of anxiety (Méndez-Ruette et al., 2019; Shi et al., 2020; Wang et al., 2022). Consistent with this, we observed increased activity in the rostral, but not caudal, insular cortex. Lastly, we observed increased activity in the ventral striatum, which corresponds to the increased avoidance as evidenced by more time on the platform (Bravo-Rivera et al., 2015; Bravo-Rivera et al., 2014; Ramirez et al., 2015). It is noteworthy that there is substantial support indicating that the ventral striatum is important for anxiety (Ahmadi et al., 2013; Bewernick et al., 2010; Burkhouse et al., 2020; Kalin et al., 2005; Xiao et al., 2021). Therefore, persistent avoidance is likely influenced by a disruption of brain regions implicated in avoidance and increases in anxiety.

We focused primarily on brain regions implicated in platform-mediated avoidance (Bravo-Rivera et al., 2015; Bravo-Rivera et al., 2014). That stated, we reasoned that altered activity in these regions of interest would be linked to reduced activity in other brain regions implicated in anxiety and avoidance, such as the hippocampus. For example, decreased activity in the basomedial amygdala has been shown to disrupt plasticity in the hippocampus (Ikegaya et al., 1996). This is important because reduced activity in the hippocampus results in dyregulation of anxiety-like and avoidance behaviors (Padilla-Coreano et al., 2019; Padilla-Coreano et al., 2016; van der Veldt et al., 2025). Future work could deeply evaluate the influence of concussion on the hippocampus and avoidance.

The extinction deficit induced by closed head injury could be rescued with pharmacological adjuncts. In support of this idea, converging reports show that some psychoactive drugs can reduce the expression of conditioned fear (Davis et al., 2025), which shares brain mechanisms with avoidance (LeDoux et al., 2017). One example is the psychostimulant yohimbine, which has been shown to reduce conditioned freezing during extinction (Hefner et al., 2008; Mueller et al., 2009). Another class of psychoactive drugs that facilitates extinction is psychedelics (Cameron et al., 2021), which reduce fear-related behaviors driven by the amygdala (Pędzich et al., 2022) and amygdala-insular connectivity (Singleton et al., 2023). Lastly, another candidate is D-cycloserine, the partial NMDA agonist that facilitates extinction of conditioned fear (Myers et al., 2011). Suffice to say, the use of any pharmacological agent around extinction of avoidance is speculative and would require cautious testing in collaboration with trained clinicians. In sum, pharmacological adjuncts could be explored to rescue extinction deficits in avoidance induced by concussion.

We did not observe changes in gut microbiota that corresponds to persistent avoidance. Ergo, changes in behavior and cellular activity in the brain are independent of the gut-microbiota-brain axis. Our lack of observation is not consistent with other work suggesting that brain injury induces gut dysbiosis (Angoa-Pérez et al., 2020; Ma et al., 2017; Treangen et al., 2018). For this reason, we provide a rationale as to why our results differ from previous reports. First, the level of injury in previous work involved moderate to severe contusion (Treangen et al., 2018) and even repeat concussions (Angoa-Pérez et al., 2020), which are more severe models of brain injury than what we used. Second, we analyzed bacterial populations from fecal pellets rather than direct analysis of gut tissue, and there is a plethora of bacteria in the mucosal lining of the gut that are not particularly represented in the fecal pellet (Tang et al., 2020). Also, we focused on only one time point after closed head injury, which was upon completion of the experiment almost two weeks after injury. This is a possible caveat, since potential dysbiosis could be transient such that bacterial levels may have returned to normal (Frankot et al., 2023; Treangen et al., 2018). Future work could sample fecal pellets at different time points, analyze subregions of the gut, and include analyses at the species level. Arguably, our lack of observed difference does not rule out the possibility that concussion induces changes in gut microbiota that correspond to persistent avoidance.

Converging lines of evidence indicate that there are sex differences in behavioral outputs in fear-related paradigms and gut microbiome analysis. In fact, female rats exhibit more “darting” behavior and less freezing than male rats as expression of learned fear (Colom-Lapetina et al., 2019). Consequently, we were unable to combine male and female rats, as their different behavioral outputs could mask any potential experimental differences. Moreover, pre-clinical models in rodents reveal sex differences in the response of the microbiome to brain injury (Baskin et al., 2023; Pasam and Dandekar, 2023; Stamper et al., 2025). Given these reasons, the present study focused on male rats. Indeed, the lack of females is a limitation of this study. Certainly, evaluation in both males and females is needed to compare the influence of concussion on avoidance and changes in microbiome for potential sex differences.

## 5. Concussion and persistent avoidance in anxiety, trauma and stress-related disorders

**Fig. 7.**
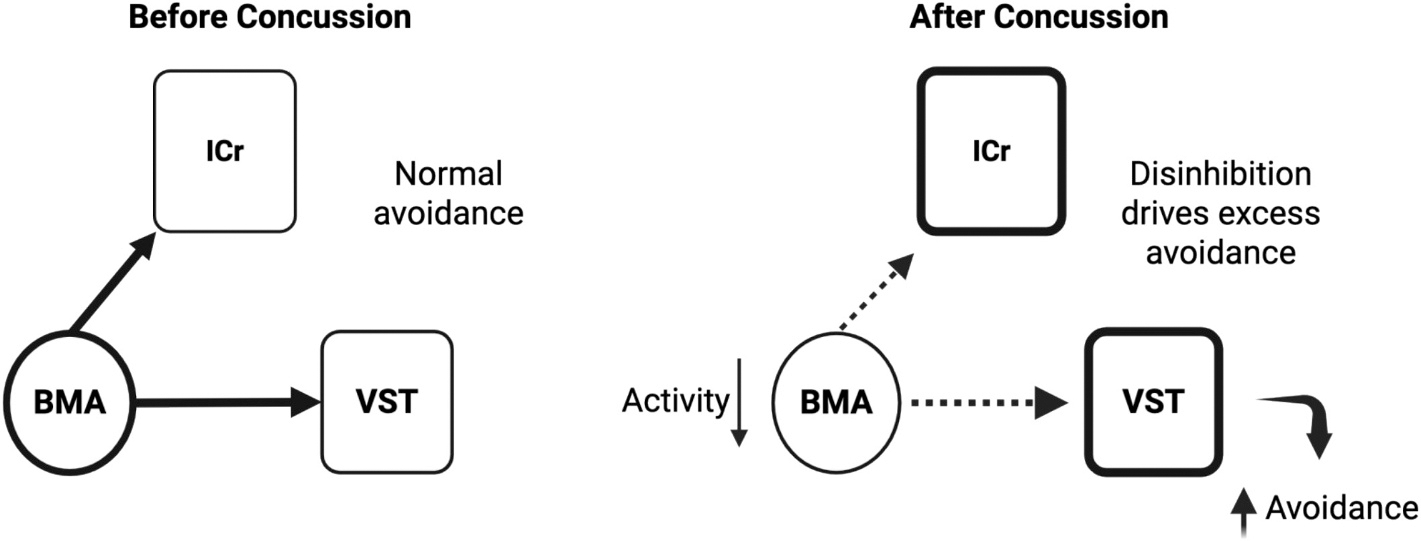
Proposed model of circuit alterations underlying increased avoidance after mild concussive brain injury. A) Under normal conditions, the basomedial amygdala (BMA) exerts modulatory control over the ventral striatum (VST) and rostral insular cortex (ICr), supporting balanced behavioral responses to threat through appropriate engagement of avoidance strategies. B) Following mild concussion, reduced activity in the BMA leads to disinhibition of its downstream targets. Thus, the ICr and VST show increased activity, potentially amplifying salience and action-related signaling. This shift in the circuit dynamic is hypothesized to bias the system toward exaggerated avoidance behavior, even in the absence of threat.

Fig. 7 posits a model by which concussion contributes to persistent avoidance. After concussion, activity is reduced in the basomedial amygdala (BMA). Since the amygdala can inhibit both the ventral striatum (VST) (Birnie et al., 2023) and rostral insular cortex (ICr) (Gil-Lievana et al., 2020; Sokolov et al., 2020; Wang et al., 2023), reduced inhibitory influence from the BMA could result in disinhibition of the VST and ICr, thereby driving avoidance.

The present study could be clinically relevant for three reasons. First, patients with brain injury display persistent avoidance of perceived threats (Labbe et al., 2014; Wood and Doughty, 2013), which is detrimental to recovery from PTSD because it impairs extinction. Second, understanding the temporal dynamics of neural changes post-injury provides insight into when interventions might be most effective regarding extinction, since patients with PTSD display impaired extinction (Milad et al., 2009; Rothbaum and Davis, 2003). Third, homologues of the basomedial amygdala, rostral insular cortex, and ventral striatum could be targeted to mitigate persistent avoidance following brain injury. To help advance research in these areas, the platform-mediated avoidance task in rodents has been incorporated into an avoidance-reward conflict task for use in non-human primates and humans (Sierra-Mercado et al., 2015a). In conclusion, understanding the intricate relationship between concussion and avoidance is crucial for developing rehabilitation strategies for mental health disorders caused or exacerbated by concussive brain injury.

## Supplementary Material

MMC5

MMC2

MMC1

MMC3

MMC4

Supplementary data to this article can be found online at https://doi.org/10.1016/j.expneurol.2026.115734.

## Data Availability

The datasets are available in public online repositories. The 16S rRNA sequencing data can be accessed from the European Nucleotide Archive (ENA) under the study accession number PRJEB108858 (ERP189693). The data are available at the following link: https://www.ebi.ac.uk/ena/browser/view/ERP189693. Specific raw behavioral data can be distributed upon request via Google Drive.

## References

[R1] Adhikari A, Lerner TN, Finkelstein J, Pak S, Jennings JH, Davidson TJ, Ferenczi E, Gunaydin LA, Mirzabekov JJ, Ye L, Kim SY, Lei A, Deisseroth K, 2015. Basomedial amygdala mediates top-down control of anxiety and fear. Nature 527, 179–185. 10.1038/nature15698. 26536109 PMC4780260

[R2] Ahmadi H, Nasehi M, Rostami P, Zarrindast MR, 2013. Involvement of the nucleus accumbens shell dopaminergic system in prelimbic NMDA-induced anxiolytic-like behaviors. Neuropharmacology 71. 10.1016/j.neuropharm.2013.03.017. 23566820

[R3] Angoa-Pérez M, Zagorac B, Anneken JH, Briggs DI, Winters AD, Greenberg JM, Ahmad M, Theis KR, Kuhn DM, 2020. Repetitive, mild traumatic brain injury results in a progressive white matter pathology, cognitive deterioration, and a transient gut microbiota dysbiosis. Sci. Rep. 10. 10.1038/s41598-020-65972-4. PMC7265445 32488168

[R4] Asmundson GJG, Stapleton JA, Taylor S, 2004. Are avoidance and numbing distinct PTSD symptom clusters? J. Trauma. Stress. 17, 467–475. 10.1007/s10960-004-5795-7. 15730065

[R5] Aupperle RL, Melrose AJ, Stein MB, Paulus MP, 2012. Executive function and PTSD: disengaging from trauma. Neuropharmacology 62, 686–694. 10.1016/j.neuropharm.2011.02.008. 21349277 PMC4719148

[R6] Baskin BM, Logsdon AF, Janet Lee S, Foresi BD, Peskind E, Banks WA, Cook DG, Schindler AG, 2023. Timing matters: sex differences in inflammatory and behavioral outcomes following repetitive blast mild traumatic brain injury. Brain Behav. Immun 110. 10.1016/j.bbi.2023.03.003. PMC10106404 36907289

[R7] Berman R, Spencer H, Boese M, Kim S, Radford K, Choi K, 2023. Loss of consciousness and righting reflex following traumatic brain injury: predictors of post-injury symptom development (a narrative review). Brain Sci. 10.3390/brainsci13050750. PMC10216326 37239222

[R8] Bewernick BH, Hurlemann R, Matusch A, Kayser S, Grubert C, Hadrysiewicz B, Axmacher N, Lemke M, Cooper-Mahkorn D, Cohen MX, Brockmann H, Lenartz D, Sturm V, Schlaepfer TE, 2010. Nucleus Accumbens deep brain stimulation decreases ratings of depression and anxiety in treatment-resistant depression. Biol. Psychiatry 67. 10.1016/j.biopsych.2009.09.013. 19914605

[R9] Birnie MT, Short AK, de Carvalho GB, Taniguchi L, Gunn BG, Pham AL, Itoga CA, Xu X, Chen LY, Mahler SV, Chen Y, Baram TZ, 2023. Stress-induced plasticity of a CRH/GABA projection disrupts reward behaviors in mice. Nat. Commun. 14. 10.1038/s41467-023-36780-x. PMC9968307 36841826

[R10] Bravo-Rivera C, Roman-Ortiz C, Brignoni-Perez E, Sotres-Bayon F, Quirk GJ, 2014. Neural structures mediating expression and extinction of platform-mediated avoidance. J. Neurosci. 34, 9736–9742. 10.1523/JNEUROSCI.0191-14.2014. 25031411 PMC4099548

[R11] Bravo-Rivera C, Roman-Ortiz C, Montesinos-Cartagena M, Quirk GJ, 2015. Persistent active avoidance correlates with activity in prelimbic cortex and ventral striatum. Front. Behav. Neurosci 9. 10.3389/fnbeh.2015.00184. PMC4502354 26236209

[R12] Bravo-Rivera H, Rubio Arzola P, Caban-Murillo A, Vélez-Avilés AN, Ayala-Rosario SN, Quirk GJ, 2021. Characterizing different strategies for resolving approach-avoidance conflict. Front. Neurosci. 15. 10.3389/fnins.2021.608922. PMC7947632 33716644

[R13] Breslau N, 2001. The epidemiology of posttraumatic stress disorder: what is the extent of the problem? J. Clin. Psychiatry 62 (Suppl. 1), 16–22. 11495091

[R14] Burkhouse KL, Jagan Jimmy, Defelice N, Klumpp H, Ajilore O, Hosseini B, Fitzgerald KD, Monk CS, Phan KL, 2020. Nucleus accumbens volume as a predictor of anxiety symptom improvement following CBT and SSRI treatment in two independent samples. Neuropsychopharmacology 45. 10.1038/s41386-019-0575-5. PMC6969163 31756730

[R15] Cáceres-Chacón M, Martínez-Guzmán O, Haddock-Martínez HA, Figueroa-Pérez A, Rodríguez-Rosado S, Suárez-Pérez J, Ramos-Sánchez RY, Godoy-Vitorino F, Sierra-Mercado D, 2025. Exposure to the herbicide glyphosate leads to inappropriate threat responses and alters gut microbial composition. Front. Toxicol. 7. 10.3389/ftox.2025.1704231 PMC12626795 41267922

[R16] Cameron LP, Tombari RJ, Lu J, Pell AJ, Hurley ZQ, Ehinger Y, Vargas MV, McCarroll MN, Taylor JC, Myers-Turnbull D, Liu T, Yaghoobi B, Laskowski LJ, Anderson EI, Zhang G, Viswanathan J, Brown BM, Tjia M, Dunlap LE, Rabow ZT, Fiehn O, Wulff H, McCorvy JD, Lein PJ, Kokel D, Ron D, Peters J, Zuo Y, Olson DE, 2021. A non-hallucinogenic psychedelic analogue with therapeutic potential. Nature 589. 10.1038/s41586-020-3008-z. PMC7874389 33299186

[R17] Carneiro CFD, Moulin TC, Macleod MR, Amaral OB, 2018. Effect size and statistical power in the rodent fear conditioning literature – a systematic review. PLoS ONE 13. 10.1371/journal.pone.0196258. PMC5919667 29698451

[R18] Celorrio M, Abellanas MA, Rhodes J, Goodwin V, Moritz J, Vadivelu S, Wang L, Rodgers R, Xiao S, Anabayan I, Payne C, Perry AM, Baldridge MT, Aymerich MS, Steed A, Friess SH, 2021. Gut microbial dysbiosis after traumatic brain injury modulates the immune response and impairs neurogenesis. Acta Neuropathol. Commun. 9, 40. 10.1186/s40478-021-01137-2. 33691793 PMC7944629

[R19] Cheng JS, Craft R, Yu G-Q, Ho K, Wang X, Mohan G, Mangnitsky S, Ponnusamy R, Mucke L, 2014. Tau reduction diminishes spatial learning and memory deficits after mild repetitive traumatic brain injury in mice. PLoS ONE 9, e115765. 25551452 10.1371/journal.pone.0115765 PMC4281043

[R20] Choi JS, Cain CK, Ledoux JE, 2010. The role of amygdala nuclei in the expression of auditory signaled two-way active avoidance in rats. Learn. Mem. 17, 139–147. 10.1101/lm.1676610. 20189958 PMC2832923

[R21] Chu C, Murdock MH, Jing D, Won TH, Chung H, Kressel AM, Tsaava T, Addorisio ME, Putzel GG, Zhou L, Bessman NJ, Yang R, Moriyama S, Parkhurst CN, Li A, Meyer HC, Teng F, Chavan SS, Tracey KJ, Regev A, Schroeder FC, Lee FS, Liston C, Artis D, 2019. The microbiota regulate neuronal function and fear extinction learning. Nature 574. 10.1038/s41586-019-1644-y. PMC6818753 31645720

[R22] Colom-Lapetina J, Li AJ, Pelegrina-Perez TC, Shansky RM, 2019. Behavioral diversity across classic rodent models is sex-dependent. Front. Behav. Neurosci 13. 10.3389/fnbeh.2019.00045. PMC6414415 30894806

[R23] Cox LM, Tatematsu BK, Guo L, LeServe DS, Mayrink J, Oliveira MG, Donnelly D, Fonseca RC, Lemos L, Lanser TB, Rosa AC, Lopes JR, Schwerdtfeger LA, Ribeiro GFC, Lobo ELC, Moreira TG, Oliveira AG, Weiner HL, Rezende RM, 2024. Gamma-delta T cells suppress microbial metabolites that activate striatal neurons and induce repetitive/compulsive behavior in mice. Brain Behav. Immun. 117. 10.1016/j.bbi.2024.01.214. 38281671

[R24] Darvas M, Fadok JP, Palmiter RD, 2011. Requirement of dopamine signaling in the amygdala and striatum for learning and maintenance of a conditioned avoidance response. Learn. Mem. 18, 136–143. 10.1101/lm.2041211. 21325435 PMC3056517

[R25] Davies DR, Olson D, Meyer DL, Scholl JL, Watt MJ, Manzerra P, Renner KJ, Forster GL, 2016. Mild traumatic brain injury with social defeat stress alters anxiety, contextual fear extinction, and limbic monoamines in adult rats. Front. Behav. Neurosci 10, 71. 27147992 10.3389/fnbeh.2016.00071 PMC4835499

[R26] Davis BJ, Rosenberg BM, Kearney MG, Treanor M, Craske MG, Barry TJ, 2025. Pharmacological enhancement of fear extinction. Trends Cogn. Sci. 10.1016/j.tics.2025.06.011. 40634208

[R27] Diehl MM, Bravo-Rivera C, Quirk GJ, 2019. The study of active avoidance: a platform for discussion. Neurosci. Biobehav. Rev. 10.1016/j.neubiorev.2019.09.010. PMC6936221 31509767

[R28] Diehl MM, Iravedra-Garcia JM, Morán-Sierra J, Rojas-Bowe G, Gonzalez-Diaz FN, Valentín-Valentín VP, Quirk GJ, 2020. Divergent projections of the prelimbic cortex bidirectionally regulate active avoidance. Elife 9. 10.7554/eLife.59281. PMC7588229 33054975

[R29] Frankot MA, O’Hearn CM, Blancke AM, Rodriguez B, Pechacek KM, Gandhi J, Hu G, Martens KM, Haar CV, 2023. Acute gut microbiome changes after traumatic brain injury are associated with chronic deficits in decision-making and impulsivity in male rats. Behav. Neurosci. 137. 10.1037/bne0000532. PMC9996537 35901372

[R30] Gennarelli TA, Thibault LE, Adams JH, Graham DI, Thompson CJ, Marcincin RP, 1982. Diffuse axonal injury and traumatic coma in the primate. Ann. Neurol. 12, 564–574. 7159060 10.1002/ana.410120611

[R31] Ghaemi M, Kheradmand D, 2025. The gut-brain axis in traumatic brain injury: literature review. J. Clin. Neurosci. 136, 111258. 10.1016/j.jocn.2025.111258. 40250160

[R32] Gil-Lievana E, Balderas I, Moreno-Castilla P, Luis-Islas J, McDevitt RA, Tecuapetla F, Gutierrez R, Bonci A, Bermúdez-Rattoni F, 2020. Glutamatergic basolateral amygdala to anterior insular cortex circuitry maintains rewarding contextual memory. Commun. Biol 3. 10.1038/s42003-020-0862-z. PMC7083952 32198461

[R33] Gonzalez A, Navas-Molina JA, Kosciolek T, McDonald D, Vázquez-Baeza Y, Ackermann G, DeReus J, Janssen S, Swafford AD, Orchanian SB, Sanders JG, Shorenstein J, Holste H, Petrus S, Robbins-Pianka A, Brislawn CJ, Wang M, Rideout JR, Bolyen E, Dillon M, Caporaso JG, Dorrestein PC, Knight R, 2018. Qiita: rapid, web-enabled microbiome meta-analysis. Nat. Methods 15, 796–798. 10.1038/s41592-018-0141-9. 30275573 PMC6235622

[R34] Grahn RE, Kalman BA, Brennan FX, Watkins LR, Maier SF, 1995. The elevated plus-maze is not sensitive to the effect of stressor controllability in rats. Pharmacol. Biochem. Behav. 52. 10.1016/0091-3057(95)00141-I. 8545475

[R35] Han W, Tellez LA, Perkins MH, Perez IO, Qu T, Ferreira J, Ferreira TL, Quinn D, Liu ZW, Gao XB, Kaelberer MM, Bohórquez DV, Shammah-Lagnado SJ, de Lartigue G, de Araujo IE, 2018. A neural circuit for gut-induced reward. Cell 175. 10.1016/j.cell.2018.08.049. 30340046

[R36] Hefner K, Hefner K, Whittle N, Whittle N, Juhasz J, Juhasz J, Norcross M, Norcross M, Karlsson RM, Karlsson RM, Saksida LM, Saksida LM, Bussey TJ, Bussey TJ, Singewald N, Singewald N, Holmes A, Holmes A, 2008. Impaired fear extinction learning and cortico-amygdala circuit abnormalities in a common genetic mouse strain. J. Neurosci. 28, 8074–8085. 18685032 10.1523/JNEUROSCI.4904-07.2008 PMC2547848

[R37] Henninger N, Dützmann S, Sicard KM, Kollmar R, Bardutzky J, Schwab S, 2005. Impaired spatial learning in a novel rat model of mild cerebral concussion injury. Exp. Neurol. 195, 447–457. 10.1016/j.expneurol.2005.06.013. 16084512

[R38] Hoban AE, Stilling RM, M Moloney G, Moloney RD, Shanahan F, Dinan TG, Cryan JF, Clarke G, 2017. Microbial regulation of microRNA expression in the amygdala and prefrontal cortex. Microbiome 5. 10.1186/s40168-017-0321-3. PMC5571609 28838324

[R39] Hoban AE, Stilling RM, Moloney G, Shanahan F, Dinan TG, Clarke G, Cryan JF, 2018. The microbiome regulates amygdala-dependent fear recall. Mol. Psychiatry 23. 10.1038/mp.2017.100. PMC5984090 28507320

[R40] Hoge CW, McGurk D, Thomas JL, Cox AL, Engel CC, Castro CA, 2008. Mild traumatic brain injury in U.S. soldiers returning from Iraq. N. Engl. J. Med. 358, 453–463. 10.1056/NEJMoa072972. 18234750

[R41] Hoge CW, Hoge CW, Castro CA, Castro CA, 2014. Treatment of generalized war-related health concerns: placing TBI and PTSD in context. JAMA 312, 1685–1686. 25335151 10.1001/jama.2014.6670

[R42] Ikegaya Y, Saito H, Abe K, 1996. The basomedial and basolateral amygdaloid nuclei contribute to the induction of long-term potentiation in the dentate gyrus in vivo. Eur. J. Neurosci. 8. 10.1111/j.1460-9568.1996.tb01327.x. 8921274

[R43] Ingram DK, de Cabo R, 2017. Calorie restriction in rodents: caveats to consider. Ageing Res. Rev. 10.1016/j.arr.2017.05.008. PMC5565679 28610949

[R44] Jiang X, Greening SG, 2021. Psychophysiological evidence for fear extinction learning via mental imagery. Psychophysiology 58. 10.1111/psyp.13906. 34287954

[R45] Kalin NH, Shelton SE, Fox AS, Oakes TR, Davidson RJ, 2005. Brain regions associated with the expression and contextual regulation of anxiety in primates. Biol. Psychiatry 58. 10.1016/j.biopsych.2005.05.021. PMC2614874 16043132

[R46] Khuman J, Khuman J, Meehan WP, Meehan WP, Zhu X, Zhu X, Qiu J, Qiu J, Hoffmann U, Hoffmann U, Zhang J, Zhang J, Giovannone E, Giovannone E, Lo EH, Lo EH, Whalen MJ, Whalen MJ, 2011. Tumor necrosis factor alpha and Fas receptor contribute to cognitive deficits independent of cell death after concussive traumatic brain injury in mice. J. Cereb. Blood Flow Metab. 31, 778–789. 20940727 10.1038/jcbfm.2010.172 PMC3049532

[R47] Kim B, Shin J, Guevarra RB, Lee Jun Hyung, Kim DW, Seol KH, Lee Ju Hoon, Kim HB, Isaacson RE, 2017. Deciphering diversity indices for a better understanding of microbial communities. J. Microbiol. Biotechnol. 27. 10.4014/jmb.1709.09027. 29032640

[R48] Klarer M, Arnold M, Günther L, Winter C, Langhans W, Meyer U, 2014. Gut vagal afferents differentially modulate innate anxiety and learned fear. J. Neurosci. 34. 10.1523/JNEUROSCI.0252-14.2014. PMC6608191 24849343

[R49] Knight R, Vrbanac A, Taylor BC, Aksenov A, Callewaert C, Debelius J, Gonzalez A, Kosciolek T, McCall LI, McDonald D, Melnik AV, Morton JT, Navas J, Quinn RA, Sanders JG, Swafford AD, Thompson LR, Tripathi A, Xu ZZ, Zaneveld JR, Zhu Q, Caporaso JG, Dorrestein PC, 2018. Best practices for analysing microbiomes. Nat. Rev. Microbiol. 10.1038/s41579-018-0029-9. 29795328

[R50] Korte SM, De Boer SF, 2003. A robust animal model of state anxiety: fear-potentiated behaviour in the elevated plus-maze. Eur. J. Pharmacol. 10.1016/S0014-2999(03)01279-2. 12600708

[R51] Labbe DR, Vance DE, Wadley V, Novack TA, 2014. Predictors of driving avoidance and exposure following traumatic brain injury. J. Head Trauma Rehabil. 29, 185–192. 23474877 10.1097/HTR.0b013e3182795211 PMC4487624

[R52] Lázaro-Muñoz G, LeDoux JE, Cain CK, 2010. Sidman instrumental avoidance initially depends on lateral and basal amygdala and is constrained by central amygdala-mediated Pavlovian processes. Biol. Psychiatry 67, 1120–1127. 10.1016/j.biopsych.2009.12.002. 20110085 PMC3085029

[R53] Leary S, Underwood W, Anthony R, Cartner S, Grandin T, Greenacre C, Gwaltney-Brant S, McCrackin MA, Meyer R, Miller D, Shearer J, Turner T, Yaning R, 2020. AVMA Guidelines for the Euthanasia of Animals: 2020 Edition* Members of the Panel on Euthanasia AVMA Staff Consultants, (Retrieved on March).

[R54] LeDoux JE, Pine DS, 2016. Using neuroscience to help understand fear and anxiety: a two-system framework. Am. J. Psychiatry. 10.1176/appi.ajp.2016.16030353. 27609244

[R55] LeDoux JE, Moscarello J, Sears R, Campese V, 2017. The birth, death and resurrection of avoidance: a reconceptualization of a troubled paradigm. Mol. Psychiatry. 10.1038/mp.2016.166. PMC5173426 27752080

[R56] Ma EL, Smith AD, Desai N, Cheung L, Hanscom M, Stoica BA, Loane DJ, Shea-Donohue T, Faden AI, 2017. Bidirectional brain-gut interactions and chronic pathological changes after traumatic brain injury in mice. Brain Behav. Immun. 66. 10.1016/j.bbi.2017.06.018. PMC5909811 28676351

[R57] Ma Y, Liu T, Fu J, Fu S, Hu C, Sun B, Fan X, Zhu J, 2019. *Lactobacillus acidophilus* exerts neuroprotective effects in mice with traumatic brain injury. J. Nutr. 149, 1543–1552. 10.1093/jn/nxz105. 31174208

[R58] Martínez JC, Cardenas F, Lamprea M, Morato S, 2002. The role of vision and proprioception in the aversion of rats to the open arms of an elevated plus-maze. Behav. Process 60. 10.1016/S0376-6357(02)00102-X. 12429388

[R59] McDonald AJ, 2020. Functional neuroanatomy of the basolateral amygdala: neurons, neurotransmitters, and circuits. In: Handbook of Behavioral Neuroscience. 10.1016/B978-0-12-815134-1.00001-5. PMC8248694 34220399

[R60] Meehan WP, Zhang J, Mannix R, Whalen MJ, 2012. Increasing recovery time between injuries improves cognitive outcome after repetitive mild concussive brain injuries in mice. Neurosurgery 71, 885–891. 10.1227/NEU.0b013e318265a439. 22743360 PMC5815628

[R61] Méndez-Ruette M, Linsambarth S, Moraga-Amaro R, Quintana-Donoso D, Méndez L, Tamburini G, Cornejo F, Torres RF, Stehberg J, 2019. The role of the rodent insula in anxiety. Front. Physiol. 10, 1–10. 10.3389/fphys.2019.00330. 30984021 PMC6450210

[R62] Meyer DL, Davies DR, Barr JL, Manzerra P, Forster GL, 2012. Mild traumatic brain injury in the rat alters neuronal number in the limbic system and increases conditioned fear and anxiety-like behaviors. Exp. Neurol. 235, 574–587. 10.1016/j.expneurol.2012.03.012. 22498103

[R63] Milad MR, Milad MR, Pitman RK, Pitman RK, Ellis CB, Ellis CB, Gold AL, Gold AL, Shin LM, Shin LM, Lasko NB, Lasko NB, Zeidan MA, Zeidan MA, Handwerger K, Handwerger K, Orr SP, Orr SP, Rauch SL, Rauch SL, 2009. Neurobiological basis of failure to recall extinction memory in posttraumatic stress disorder. Biol. Psychiatry 66, 1075–1082. 19748076 10.1016/j.biopsych.2009.06.026 PMC2787650

[R64] Moscarello JM, LeDoux JE, 2013. Active avoidance learning requires prefrontal suppression of amygdala-mediated defensive reactions. J. Neurosci. 10.1523/JNEUROSCI.2596-12.2013. PMC3607300 23447593

[R65] Mowrer O, Lamoreaux R, 1946. Fear as an intervening variable in avoidance conditioning. J. Comp. Psychol. 39, 29–50. 10.1037/h0060150. 21018303

[R66] Mueller D, Olivera-Figueroa LA, Pine DS, Quirk GJ, 2009. The effects of yohimbine and amphetamine on fear expression and extinction in rats. Psychopharmacology 204. 10.1007/s00213-009-1491-x. PMC2692542 19242678

[R67] Mueller D, Bravo-Rivera C, Quirk GJ, 2010. Infralimbic D2 receptors are necessary for fear extinction and extinction-related tone responses. Biol. Psychiatry 68, 1055–1060. 20926066 10.1016/j.biopsych.2010.08.014 PMC2981677

[R68] Mychasiuk R, Farran A, Esser MJ, 2014. Assessment of an experimental rodent model of pediatric mild traumatic brain injury. J. Neurotrauma 31, 749–757. 10.1089/neu.2013.3132. 24283269

[R69] Myers KM, Carlezon WA, Davis M, 2011. Glutamate receptors in extinction and extinction-based therapies for psychiatric illness. Neuropsychopharmacology. 10.1038/npp.2010.88. PMC2994960 20631689

[R70] Needham BD, Funabashi M, Adame MD, Wang Z, Boktor JC, Haney J, Wu WL, Rabut C, Ladinsky MS, Hwang SJ, Guo Y, Zhu Q, Griffiths JA, Knight R, Bjorkman PJ, Shapiro MG, Geschwind DH, Holschneider DP, Fischbach MA, Mazmanian SK, 2022. A gut-derived metabolite alters brain activity and anxiety behaviour in mice. Nature 602. 10.1038/s41586-022-04396-8. PMC9170029 35165440

[R71] Padilla-Coreano N, Bolkan SS, Pierce GM, Blackman DR, Hardin WD, Garcia-Garcia AL, Spellman TJ, Gordon JA, 2016. Direct ventral hippocampal-prefrontal input is required for anxiety-related neural activity and behavior. Neuron 89. 10.1016/j.neuron.2016.01.011. PMC4760847 26853301

[R72] Padilla-Coreano N, Canetta S, Mikofsky RM, Alway E, Passecker J, Myroshnychenko MV, Garcia-Garcia AL, Warren R, Teboul E, Blackman DR, Morton MP, Hupalo S, Tye KM, Kellendonk C, Kupferschmidt DA, Gordon JA, 2019. Hippocampal-prefrontal theta transmission regulates avoidance behavior. Neuron 104. 10.1016/j.neuron.2019.08.006. PMC6842114 31521441

[R73] Palmer CP, Metheny HE, Elkind JA, Cohen AS, 2016. Diminished amygdala activation and behavioral threat response following traumatic brain injury. Exp. Neurol. 277, 215–226. 10.1016/j.expneurol.2016.01.004. 26791254 PMC4761321

[R74] Pasam T, Dandekar MP, 2023. Fecal microbiota transplantation unveils sex-specific differences in a controlled cortical impact injury mouse model. Front. Microbiol. 14. 10.3389/fmicb.2023.1336537. PMC10894955 38410824

[R75] Paxinos G, Watson Charles, 2007. The Rat Brain in Stereotaxic Coordinates Sixth Edition. Elsevier Academic Press.

[R76] Pędzich BD, Rubens S, Sekssaoui M, Pierre A, Van Schuerbeek A, Marin P, Bockaert J, Valjent E, Bécamel C, De Bundel D, 2022. Effects of a psychedelic 5-HT2A receptor agonist on anxiety-related behavior and fear processing in mice. Neuropsychopharmacology 47. 10.1038/s41386-022-01324-2. PMC9117291 35449450

[R77] Plendl W, Wotjak CT, 2010. Dissociation of within-and between-session extinction of conditioned fear. J. Neurosci. 30. 10.1523/JNEUROSCI.6038-09.2010. PMC6632794 20371819

[R78] Quirk GJ, Russo GK, Barron JL, Lebron K, 2000. The role of ventromedial prefrontal cortex in the recovery of extinguished fear. J. Neurosci. 20, 6225–6231. 10934272 10.1523/JNEUROSCI.20-16-06225.2000 PMC6772571

[R79] Ramirez F, Moscarello JM, Le Doux JE, Sears RM, 2015. Active avoidance requires a serial basal amygdala to nucleus accumbens shell circuit. J. Neurosci. 35, 3470–3477. 10.1523/JNEUROSCI.1331-14.2015. 25716846 PMC4339356

[R80] Reger ML, Poulos AM, Buen F, Giza CC, Hovda DA, Fanselow MS, 2012. Concussive brain injury enhances fear learning and excitatory processes in the amygdala. Biol. Psychiatry 71, 335–343. 10.1016/j.biopsych.2011.11.007. 22169439 PMC3264758

[R81] Rice MW, Pandya JD, Shear DA, 2019. Gut microbiota as a therapeutic target to ameliorate the biochemical, neuroanatomical, and behavioral effects of traumatic brain injuries. Front. Neurol 10, 875. 10.3389/fneur.2019.00875. 31474930 PMC6706789

[R82] Rosas-Vidal LE, Lozada-Miranda V, Cantres-Rosario Y, Vega-Medina A, Melendez L, Quirk GJ, 2018. Alteration of BDNF in the medial prefrontal cortex and the ventral hippocampus impairs extinction of avoidance. Neuropsychopharmacology 43. 10.1038/s41386-018-0176-8. PMC6224579 30127343

[R83] Rothbaum BO, Davis M, 2003. Applying learning principles to the treatment of post-trauma reactions. Ann. N. Y. Acad. Sci. 1008, 112–121. 14998877 10.1196/annals.1301.012

[R84] Shannon CE, 1948. A mathematical theory of communication. Bell Syst. Tech. J 27. 10.1002/j.1538-7305.1948.tb01338.x.

[R85] Shi T, Feng S, Wei M, Zhou W, 2020. Role of the anterior agranular insular cortex in the modulation of fear and anxiety. Brain Res. Bull. 155. 10.1016/j.brainresbull.2019.12.003. 31816406

[R86] Shin LM, Liberzon I, 2010. The neurocircuitry of fear, stress, and anxiety disorders. Neuropsychopharmacology 35, 169–191. 10.1038/npp.2009.83. 19625997 PMC3055419

[R87] Sierra-Mercado D, Corcoran KA, Lebrón-Milad K, Quirk GJ, 2006. Inactivation of the ventromedial prefrontal cortex reduces expression of conditioned fear and impairs subsequent recall of extinction. Eur. J. Neurosci. 24, 1751–1758. 17004939 10.1111/j.1460-9568.2006.05014.x

[R88] Sierra-Mercado D, Padilla-Coreano N, Quirk GJ, 2011. Dissociable roles of prelimbic and infralimbic cortices, ventral hippocampus, and basolateral amygdala in the expression and extinction of conditioned fear. Neuropsychopharmacology 36, 529–538. 10.1038/npp.2010.184. 20962768 PMC3005957

[R89] Sierra-Mercado D, Deckersbach T, Arulpragasam AR, Chou T, Rodman AM, Duffy A, McDonald EJ, Eckhardt CA, Corse AK, Kaur N, Eskandar EN, Dougherty DD, 2015a. Decision making in avoidance-reward conflict: a paradigm for non-human primates and humans. Brain Struct. Funct. 220, 2509–2517. 24969127 10.1007/s00429-014-0796-7

[R90] Sierra-Mercado D, McAllister LM, Lee CCH, Milad MR, Eskandar EN, Whalen MJ, 2015b. Controlled cortical impact before or after fear conditioning does not affect fear extinction in mice. Brain Res. 1606, 133–141. 10.1016/j.brainres.2015.02.031. 25721797 PMC4518729

[R91] Simon DW, Rogers MB, Gao Y, Vincent G, Firek BA, Janesko-Feldman K, Vagni V, Kochanek PM, Ozolek JA, Mollen KP, Clark RSB, Morowitz MJ, 2020. Depletion of gut microbiota is associated with improved neurologic outcome following traumatic brain injury. Brain Res. 1747, 147056. 10.1016/j.brainres.2020.147056. 32798452 PMC7521107

[R92] Singleton SP, Wang JB, Mithoefer M, Hanlon C, George MS, Mithoefer A, Mithoefer O, Coker AR, Yazar-Klosinski B, Emerson A, Doblin R, Kuceyeski A, 2023. Altered brain activity and functional connectivity after MDMA-assisted therapy for post-traumatic stress disorder. Front. Psychol. 13. 10.3389/fpsyt.2022.947622. PMC9879604 36713926

[R93] Sokolov AA, Zeidman P, Erb M, Pollick FE, Fallgatter AJ, Ryvlin P, Friston KJ, Pavlova MA, 2020. Brain circuits signaling the absence of emotion in body language. Proc. Natl. Acad. Sci. USA 117. 10.1073/pnas.2007141117. PMC7456113 32764147

[R94] Stamper CE, Cominski TP, Hoisington AJ, Yoe CW, Agbolou XM, Stiritz VA, Interian A, Goodman M, Hazlett EA, Myers CE, Beck KD, Brenner LA, 2025. Longitudinal effects of mild traumatic brain injury on the gut microbiome and acoustic startle response in male and female rats. J. Neurotrauma. 10.1177/08977151251372118. PMC12504221 40899103

[R95] Stout DM, Acheson DT, Moore TM, Gur RC, Baker DG, Geyer MA, Risbrough VB, 2018. Individual variation in working memory is associated with fear extinction performance. Behav. Res. Ther 102. 10.1016/j.brat.2018.01.002. PMC6182776 29331727

[R96] Su X, 2021. Elucidating the beta-diversity of the microbiome: from global alignment to local alignment. mSystems 6. 10.1128/msystems.00363-21. PMC8409727 34402645

[R97] Tang Q, Jin G, Wang G, Liu T, Liu X, Wang B, Cao H, 2020. Current sampling methods for gut microbiota: a call for more precise devices. Front. Cell. Infect. Microbiol. 10.3389/fcimb.2020.00151. PMC7161087 32328469

[R98] Taylor BC, Taylor BC, Hagel EM, Hagel EM, Carlson KF, Carlson KF, Cifu DX, Cifu DX, Cutting A, Cutting A, Bidelspach DE, Bidelspach DE, Sayer NA, Sayer NA, 2012. Prevalence and costs of co-occurring traumatic brain injury with and without psychiatric disturbance and pain among Afghanistan and Iraq war veteran V.A. Users. Med. Care 50, 342–346. 22228249 10.1097/MLR.0b013e318245a558

[R99] Treangen TJ, Wagner J, Burns MP, Villapol S, 2018. Traumatic brain injury in mice induces acute bacterial dysbiosis within the fecal microbiome. Front. Immunol. 9. 10.3389/fimmu.2018.02757. PMC6278748 30546361

[R100] van der Veldt S, Bagot RC, Ciocchi S, Ito R, Kheirbek MA, MacAskill AF, Amilhon B, 2025. Anxiety and beyond: diversity in ventral Hippocampus circuits and function. J. Neurosci. 10.1523/JNEUROSCI.1304-25.2025. PMC12614051 41224652

[R101] Wang Qi, Zhu JJ, Wang L, Kan YP, Liu YM, Wu YJ, Gu X, Yi X, Lin ZJ, Wang Qin, Lu JF, Jiang Q, Li Y, Liu MG, Xu NJ, Zhu MX, Wang LY, Zhang S, Li WG, Le Xu T, 2022. Insular cortical circuits as an executive gateway to decipher threat or extinction memory via distinct subcortical pathways. Nat. Commun. 13. 10.1038/s41467-022-33241-9. PMC9492683 36130959

[R102] Wang L, Hu X, Ren Y, Lv J, Zhao S, Guo L, Liu T, Han J, 2023. Arousal modulates the amygdala-insula reciprocal connectivity during naturalistic emotional movie watching. Neuroimage 279. 10.1016/j.neuroimage.2023.120316. 37562718

[R103] Watanabe M, Uematsu A, Johansen JP, 2021. Enhanced synchronization between prelimbic and infralimbic cortices during fear extinction learning. Mol. Brain 14. 10.1186/s13041-021-00884-6. PMC8666018 34895283

[R104] Weber JT, 2007. Experimental models of repetitive brain injuries. Prog. Brain Res. 10.1016/S0079-6123(06)61018-2. 17618983

[R105] Witgen BM, Lifshitz J, Smith ML, Schwarzbach E, Liang S-L, Grady MS, Cohen AS, 2005. Regional hippocampal alteration associated with cognitive deficit following experimental brain injury: a systems, network and cellular evaluation. Neuroscience 133, 1–15. 10.1016/j.neuroscience.2005.01.052. 15893627

[R106] Wood RL, Doughty C, 2013. Alexithymia and avoidance coping following traumatic brain injury. J. Head Trauma Rehabil. 28, 98–105. 22495103 10.1097/HTR.0b013e3182426029

[R107] Wu L, Kalish BT, Finander B, Cao T, Jin G, Yahya T, Levy ES, Kukreja B, LaRovere ES, Chung JY, Lo EH, Brown-Whalen A, El Khoury J, Kaplan DL, Whalen MJ, 2022. Repetitive mild closed head injury in adolescent mice is associated with impaired proteostasis, neuroinflammation, and tauopathy. J. Neurosci. 42. 10.1523/JNEUROSCI.0682-21.2021. PMC8944232 35105673

[R108] Xiao Q, Zhou X, Wei P, Xie L, Han Y, Wang J, Cai A, Xu F, Tu J, Wang L, 2021. A new GABAergic somatostatin projection from the BNST onto accumbal parvalbumin neurons controls anxiety. Mol. Psychiatry 26. 10.1038/s41380-020-0816-3. PMC8589681 32555286

[R109] Zhang L, Xu H, Ding N, Li X, Chen X, Chen Z, 2021. Beneficial effects on brain micro-environment by caloric restriction in alleviating neurodegenerative diseases and brain aging. Front. Physiol. 10.3389/fphys.2021.715443. PMC8660583 34899367

[R110] Zhao J, Huynh J, Hylin MJ, O’Malley JJ, Perez A, Moore AN, Dash PK, 2018. Mild traumatic brain injury reduces spine density of projection neurons in the medial prefrontal cortex and impairs extinction of contextual fear memory. J. Neurotrauma 35, 149–156. 10.1089/neu.2016.4898. 28665166 PMC5757078

