# Supplementary material for: Mild concussion impairs extinction of avoidance and alters respective brain circuits in male rats
Source: Exp Neurol. Author manuscript; Available in PMC 2026 May 27. (PMC13215635; doi:10.1016/j.expneurol.2026.115734)
Supplement: MMC5 [file NIHMS2159082-supplement-MMC5.pdf]

Supplementary Information

The premise of the study included to determine how closed head injury influences the interaction of brain regions implicated in avoidance. Thus, after bar press training, all rats underwent surgery for infusion of anatomical tracers. Anesthesia was achieved using isoflurane (4-5%) in an induction chamber. Next, rats were placed in a stereotaxic apparatus (Kopf Instruments, Tujunga, CA). Anesthesia was maintained by isoflurane (2-3%) via face mask. In one cohort (n=24), rats were unilaterally infused with the tracer cholera-toxin B (CTB; 0.25 μL) into the basolateral amygdala (AP: -2.76 mm; ML: 4.70 mm; DV: 8.90 mm). In a separate cohort (n=22), rats were infused with the tracer fast blue (0.20 μL) into the prelimbic cortex (AP: 2.64 mm; ML: 0.6 mm; DV: 4.2 mm). The wound was closed with tissue glue (Medbond, Stoelting, Wood Dale, IL) and triple antibiotic was placed on the wound suture. Lastly, meloxicam (5mg/kg, I.M.) was administered. Two rats died during surgery. All other rats recovered from surgery for two weeks prior to any behavioral assessments. Notably, analysis of the anatomical tracers was not included in the present study due to technical limitations.
