# Supplementary figures and images for: Mild concussion impairs extinction of avoidance and alters respective brain circuits in male rats
Source: Exp Neurol. Author manuscript; Available in PMC 2026 May 27. (PMC13215635; doi:10.1016/j.expneurol.2026.115734)

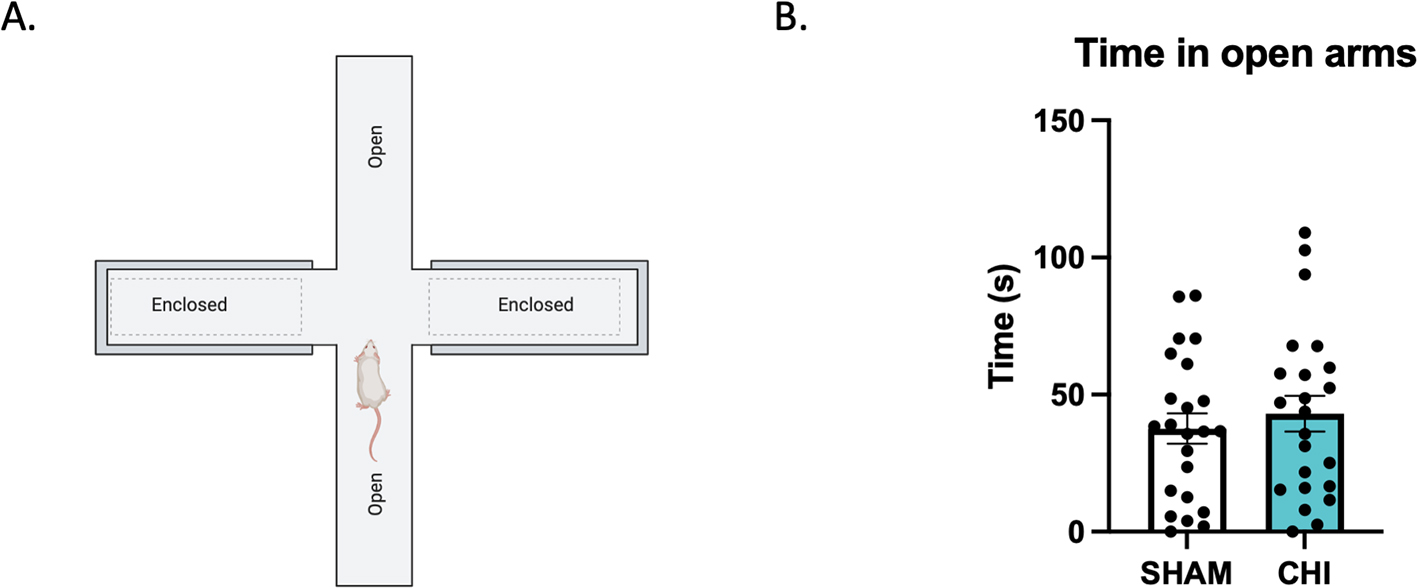

Supplement: MMC2 [file NIHMS2159082-supplement-MMC2.jpg]

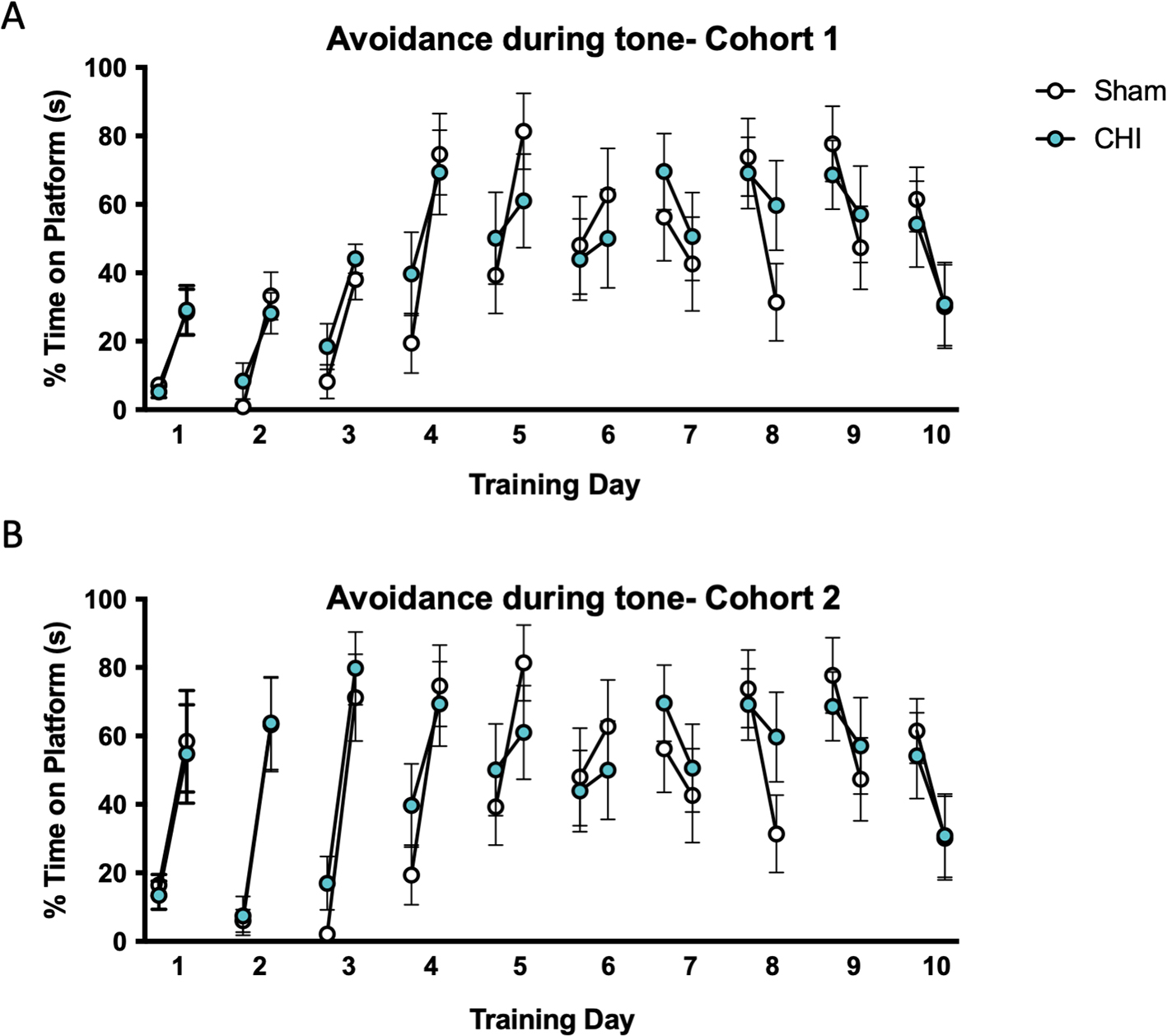

Supplement: MMC1 [file NIHMS2159082-supplement-MMC1.jpg]
